# GLP‐2 and GIP acutely increase superior mesenteric artery blood flow in male rats, and the effect is independent of nitric oxide and vasoactive intestinal peptide

**DOI:** 10.14814/phy2.70699

**Published:** 2025-12-13

**Authors:** Katrine D. Galsgaard, Bolette Hartmann, Mette M. Rosenkilde, Jens J. Holst, Lærke S. Gasbjerg, Charlotte M. Sørensen

**Affiliations:** ^1^ Department of Biomedical Sciences, Faculty of Health and Medical Sciences University of Copenhagen Copenhagen Denmark; ^2^ Novo Nordisk Foundation Center for Basic Metabolic Research, Faculty of Health and Medical Sciences University of Copenhagen Copenhagen Denmark

**Keywords:** blood flow, blood pressure, GIP, GLP‐2, superior mesenteric artery

## Abstract

Following a meal, splanchnic blood flow increases. This is important for nutrient absorption and is regulated by the enteric nervous system and gastrointestinal (GI) hormones. Specifically, postprandial release of two GI hormones—glucagon‐like peptide‐2 (GLP‐2) and glucose‐dependent insulinotropic polypeptide (GIP) has been implicated in the regulation of splanchnic blood flow. We investigated the acute effects of GLP‐2 and GIP alone or in combination on superior mesenteric artery blood flow in anesthetized rats using a transit‐time flow probe and potential mediators of the GLP‐2 and GIP effects using a nitric oxide (NO) synthase inhibitor and a vasoactive intestinal peptide (VIP) receptor antagonist. We also investigated the effects of a newly developed (human) GIP/GLP‐2 receptor co‐agonist and the effects of the GLP‐2 receptor antagonist, GLP‐2(3–33). Both GLP‐2, GIP, and the co‐agonist acutely increased superior mesenteric artery blood flow in anesthetized rats. The increase in blood flow was independent of the NO synthase inhibitor and VIP receptor antagonism. A synergistic effect of GLP‐2 and GIP combined, or the GIP/GLP‐2 receptor co‐agonist, could not be demonstrated. GLP‐2(3–33) effectively antagonized the GLP‐2‐induced increase in superior mesenteric artery blood flow. Our results establish GLP‐2 and GIP as potent stimulators of superior mesenteric artery blood flow in anesthetized rats.

## Graphical abstract

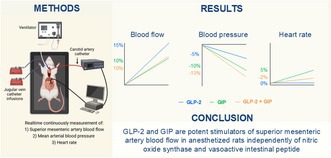

## 1 INTRODUCTION

Following a meal, blood flow is redistributed to the splanchnic area (the gastrointestinal (GI) tract and liver) from the remaining reservoirs (Someya et al., 2008). This postprandial increase in splanchnic blood flow ensures that the ingested nutrients are adequately absorbed and distributed throughout the body. GI hormones, including glucagon‐like peptide‐1 (GLP‐1), glucagon‐like peptide‐2 (GLP‐2), glucose‐dependent insulinotropic polypeptide (GIP), and Peptide YY, are released during a meal. In concert with the enteric nervous system, these hormones are suggested to regulate nutrient intake, absorption, digestion, and splanchnic blood flow (Bergmann et al., 2019; Koffert et al., 2017; Okazaki et al., 1986). Specifically, postprandial secretion of GLP‐2 and GIP has been proposed as an important stimulus for increasing splanchnic blood flow.

GLP‐2 administration increases superior mesenteric artery blood flow (supplying the duodenum, jejunum, ileum, and a part of the proximal colon) in healthy humans (Bremholm et al., 2009, 2010), patients with short bowel syndrome (Bremholm et al., 2011; Høyerup et al., 2013), calves (Taylor‐Edwards et al., 2011), anesthetized rats (Deniz et al., 2007), and piglets on total parenteral nutrition (Guan et al., 2006; Stephens et al., 2006). The GLP‐2 mediated increase in blood flow is rapid (<10 min) and is thought to be restricted to the superior mesenteric artery (Bremholm et al., 2010; Deniz et al., 2007; Guan et al., 2003; Stephens et al., 2006). In pigs, GLP‐2 also increases blood flow in the portal vein (Guan et al., 2003). The GLP‐2 receptor is localized to subepithelial myofibroblasts and enteric neurons in the small intestine and colon in both rodents and humans (Bjerknes & Cheng, 2001; Ørskov et al., 2005; Yusta et al., 2000), suggesting that an indirect mechanism is involved in the regulation of blood flow. GLP‐2 receptors co‐localize with endothelial nitric oxide (NO) synthase (eNOS) protein and are also found in vasoactive intestinal peptide (VIP) containing enteric neurons in the GI tract (Guan et al., 2006). In mice, GLP‐2 receptor expression is localized to enteric neurons and lamina propria stromal cells in the gut, and vascular GLP‐2 receptor expression is localized to mesenteric veins and not arteries (Yusta et al., 2019). Furthermore, GLP‐2 administration increases eNOS and VIP expression in rodent enteric neurons (de Heuvel et al., 2012). Since the NO synthase inhibitor *L‐NG‐nitroarginine methyl ester* (L‐NAME) lowers the GLP‐2‐induced increase in blood flow in pigs on total parenteral nutrition and anesthetized rats, the effect is suggested to be NO dependent (Deniz et al., 2007; Guan et al., 2003).

GIP has been shown to enhance blood flow to peripheral adipose tissue (Fara & Salazar, 1978) and to increase superior mesenteric artery blood flow dose‐dependently in anesthetized cats (Fara & Salazar, 1978) and dogs (Kogire et al., 1988) (in dogs, also blood flow through the portal vein (Kogire et al., 1992)). Moreover, GIP increases jejunal perfusion (Honka et al., 2017; Koffert et al., 2017) and superior mesenteric artery and portal vein blood flow in healthy humans (Rasmussen et al., 2025). The GIP receptor is thought to be expressed in endothelial cells, as GIP receptor mRNA has been detected in human endothelial cell lines from various vascular beds (Ojima et al., 2012; Zhong et al., 2000).

The mechanisms by which GLP‐2 and GIP increase splanchnic blood flow, however, remain uncertain, and the combined effect of GLP‐2 and GIP has not been investigated. Additionally, the specific effects of GIP on intestinal parameters have been overshadowed by its insulinotropic effects. However, the recent implementations of GLP‐1/GIP receptor co‐agonists in the treatment of metabolic diseases (Jastreboff et al., 2022; Rosenstock et al., 2021) and GLP‐2/GIP receptor co‐agonists for the treatment of bone disorders (Gabe, Skov‐Jeppesen, et al., 2022) have made it important to study these presumably prominent effects of GIP. Here, we tested the hypothesis that GLP‐2 and GIP, when combined, exert a synergistic stimulatory effect on superior mesenteric artery blood flow. Here, we used native hormones as well as a co‐agonist (Co‐Ago) derived from the GIP‐GLP‐2 receptor co‐agonists described previously (Gabe, Skov‐Jeppesen, et al., 2022). We further investigated whether the effect of GLP‐2 and GIP is mediated through NO or VIP, and if the truncated GLP‐2 peptide GLP‐2(3–33) can antagonize the GLP‐2‐induced changes in blood flow in our rat model. Establishing the effect and underlying mechanisms of GLP‐2, GIP, and GLP‐2 + GIP on blood flow to the GI tract is important for the understanding of GI hormone physiology and regulation of splanchnic blood flow, as well as their importance for the postprandial absorptive processes.

## 2 METHODS

### 2.1 Animal studies

All animal studies were approved by the Danish Animal Experiments Inspectorate (2020‐15‐0201‐00547) and the local ethical committee (Department of Experimental Medicine). The rats followed a light cycle of 12 h (lights on 6 am to 6 pm) and were housed in groups of two to four in individually ventilated cages with free access to water and standard chow (Scientific diets, SAFE® D30).

### 2.2 Experimental protocol

The experiments were performed in male non‐fasted Sprague Dawley rats (weighing 290–420 g) obtained from Taconic, Lille Skensved, Denmark (except for the experiments presented in Figures S3 and S5 where one and two male Wistar rats were used, respectively). Anesthesia was induced with isoflurane (induction dose 5% and maintenance dose 2%–2.5%) delivered in 35% oxygen and 65% nitrogen. Two polyethylene catheters were placed in the right jugular vein for infusions, and one catheter was placed in the left carotid artery for continuous measurement of the systemic blood pressure and heart rate by a Statham P23‐dB pressure transducer (Gould, Oxnard, CA). A tracheostomy was performed, and the rat was connected to, and ventilated by, an animal ventilator (Ugo Basile, Italy; 70 breaths/min). The rat was placed on a heating table to maintain its body temperature at 37°C. An intravenous (i.v.) bolus injection of the muscle relaxant cisatracurium besilate (Nimbex; GlaxoSmithKline, Brøndby, Denmark) in 0.5 mL 0.9% saline was administered, followed by a continuous i.v. infusion of 0.6 mg/mL at 20 μL/min. Additional saline was continuously infused at 20 μL/min throughout the experiment. A midline incision opened the abdomen, and the superior mesenteric artery was exposed, and an ultrasonic flow probe (Transonic 1PRB) was placed around the artery to measure blood flow. Rats were left undisturbed for 30 min after the surgical procedures before the initiation of the experiment.

Native human GLP‐2(1–33) (H‐7742, Bachem, Bubendorf, Switzerland) was dissolved in a 50 mM sodium hydrogen carbonate buffer, pH 8.5, containing 0.5% human serum albumin and NaCl 9 mg/mL. Human GIP(1–42) (PolyPeptide group, Strasbourg, France) was dissolved in 0.9% NaCl, containing in addition 0.5% human serum albumin. Both peptide solutions had a purity above 97%. GLP‐2(3–33) (Caslo, Aps, Denmark) was dissolved in a 10 mM sodium hydrogen carbonate buffer, pH 8.5, containing in addition 0.5% human serum albumin and NaCl 9 mg/mL. Human GIP(1–42), human GLP‐2(1–33), and GLP‐2(3–33) solutions were prepared by the Capital Region Pharmacy (Herlev, Denmark). The GLP‐2‐ and GIP‐receptor co‐agonist was kindly provided by Bainan Biotech, Copenhagen (Gabe, Skov‐Jeppesen, et al., 2022). Before the experiments, the peptides were additionally dissolved in 0.9% saline +1% bovine serum albumin (BSA, Sigma‐Aldrich cas no. 9048‐46‐8) to achieve the desired concentrations. Peptides were given as an i.v. bolus injection (100 μL) in the jugular catheter used for the continuous saline infusion. The catheter was flushed with app. 100 μL saline after each bolus injection. As a negative control, an i.v. bolus injection of 100 μL 0.9% saline or 0.9% saline +1% BSA was given. The NO synthase inhibitor L‐NAME (Merck, cas no. 51298‐62‐5) was dissolved in 0.9% saline to a concentration of 2.5 mg/mL and administered continuously at a rate of 20 μL/min in the jugular catheter used for the continuous saline infusion. The VIP hybrid antagonist neurotensin(6–11)VIP(7–28), a competitive antagonist of VIP‐binding to its receptors (VIPR‐An, Bachem, cas no. 125093‐93‐8), was dissolved in 0.9% saline to a concentration of 0.1 mg/mL and administered continuously at a rate of 20 μL/min in the jugular catheter used for the continuous saline infusion. GLP‐2(3–33) was administered continuously at a concentration of 700 μg/mL and at a rate of 20 μL/min in the jugular catheter used for the continuous saline infusion.

The experimental interventions within each rat were performed in a randomized order with the exception of experiments investigating the effects of L‐NAME, VIPR‐An, and GLP‐2(3–33).

### 2.3 In vitro pharmacological characterization

#### 2.3.1 Transfections and tissue culture

COS‐7 cells (ATCC, Virginia, USA) were cultured at 10% CO_2_ and 37°C in DMEM 1885 supplemented with 10% FBS, 2 mmol/L glutamine, 180 units × mL^−1^ penicillin and 45 g × mL^−1^ streptomycin. Transient transfection was performed using the calcium phosphate precipitation method with the addition of chloroquine (cat. no. 50‐63‐5, Sigma Aldrich) (Kissow et al., 2012). cAMP assay: COS‐7 cells, transiently transfected with the rat GIP receptor or GLP‐2 receptor, were seeded out in white 96‐well plates at a density of 3 × 10^4^ cells per well. The day after, the cells were washed twice with HEPES‐buffered saline (HBS) buffer and incubated with HBS and 1 mmol/L IBMX for 30 min at 37°C. The native hormones and the co‐agonist were added and incubated for 30 min at 37°C. Rat GIP and GLP‐2 were custom‐made from Wuxi with a purity of >95%. The HitHunterTM cAMP XS assay (cat. no. 90‐0075SM2, DiscoveRx, Herlev, Denmark) was carried out according to the manufacturer's instructions.

### 2.4 Statistics

To evaluate the effects of the bolus injections on blood flow, mean arterial blood pressure, and heart rate, recordings were made every second, and values were extracted every 10th second from 1 min before the bolus injection until 2 min after the bolus injection. The post‐injection recordings shown in the line graphs throughout the manuscript are expressed as % changes from baseline, calculated as ((stimulation−baseline)/baseline) × 100%. The stimulation is the actual value extracted every 10th second up to 2 min after the bolus injection. The baseline is the mean of the actual values extracted every 10th second for 1 min before the bolus injection. The bar graphs show the effect of the bolus injections calculated as follows: ((stimulation−baseline)/baseline) × 100%. The baseline is the mean of the actual values extracted every 10th s 1 min before the bolus injection. The stimulation is the mean of the actual values extracted every 10th s up to 2 min after the bolus injection. All actual values are shown in the Figures S1–S18. In the experiments investigating the effects of the VIP receptor antagonist (VIPR‐An) and GLP‐2(3–33), values were extracted every 10th s until 5 min after the bolus injection. Due to technical issues, the heart rate data could not be obtained in some of the experiments. Groups were compared by one‐way ANOVA or mixed effects analysis corrected for multiple comparisons using the Dunnett or Tukey test, or unpaired *t*‐test. In the figures, significant differences are reported using asterisks where *p < 0.5, **p < 0.01, ***p < 0.001, ****p < 0.0001.

For all experiments, *p* < 0.05 was considered significant. Data are shown as mean ± SD unless otherwise stated. All statistical analyses were done in GraphPad Prism version 10.2.0 (La Jolla, California, USA).

## 3 RESULTS

### 3.1 In vitro pharmacological characterization of GLP‐2(1–33), GIP(1–42), and the GLP‐2/GIP receptor co‐agonist (Co‐Ago) on rat GIP and GLP‐2 receptors

**FIGURE 1:**
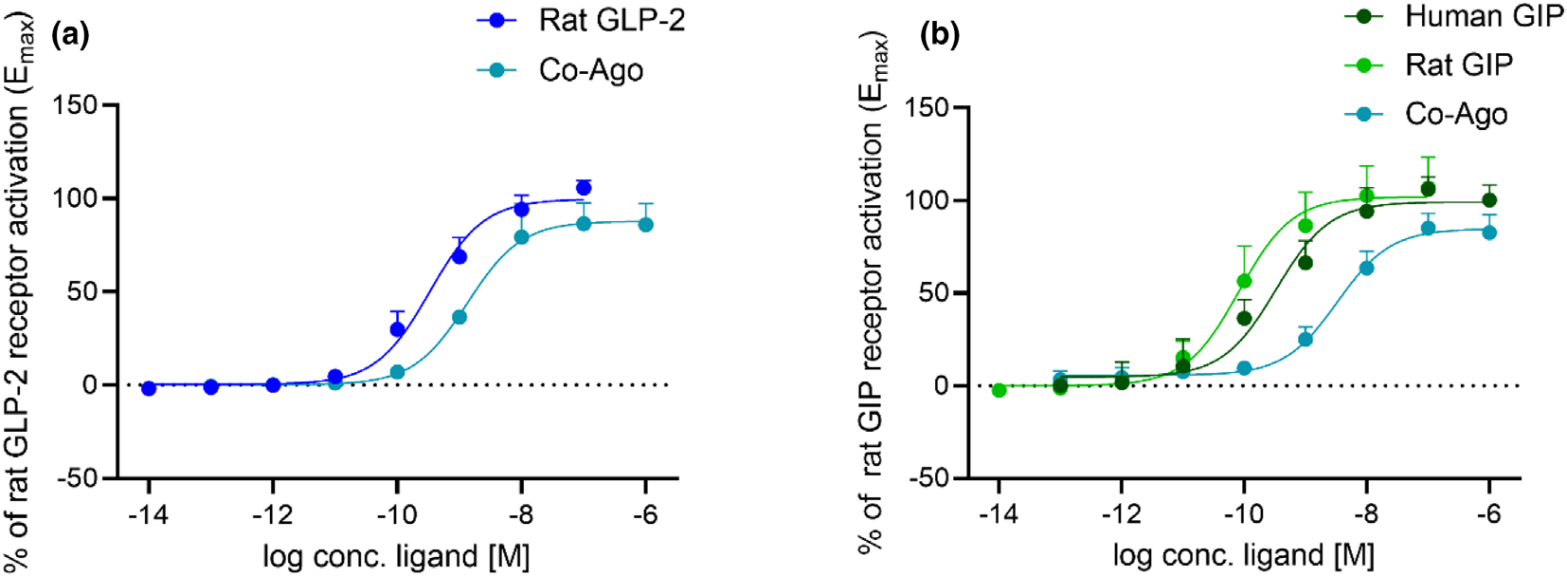
In vitro pharmacological characterization of GLP‐2(1–33), GIP(1–42), and the GLP‐2/GIP receptor co‐agonist (Co‐Ago) on their respective rat receptors. (a) Co‐Ago on the rat GLP‐2 receptor compared to rat GLP‐2 (*n* = 3 and 6, respectively), (b) human GIP and the GIP/GLP‐2 receptor co‐agonist (Co‐Ago) on the rat GIP receptor compared to rat GIP (*n* = 5, *n* = 3, and *n* = 9, respectively). (a) and (b) show dose–response relationships and the readout is cAMP accumulation in transiently transfected COS‐7 cells. Data shown as mean ± SD.

We used the native human peptides, GLP‐2(1–33) and GIP(1–42), to investigate the effects of GLP‐2 and GIP in rats. Therefore, we initially tested the effect of the human peptides on their respective rat receptors. Human GLP‐2 has been shown to activate the rat GLP‐2 receptor with a potency similar to that of rat GLP‐2 (LogEC_50_ = −9.7 ± 0.03 (Gadgaard et al., 2023) and LogEC_50_ = −9.5 ± 0.1, respectively, the latter shown in Figure 1a). In contrast, human GIP activated the rat GIP receptor with a slightly lower potency than that of rat GIP (LogEC_50_ = −9.5 ± 0.1 and LogEC_50_ = −10.1 ± 0.1, respectively) (Figure 1b), consistent with previous data (Sparre‐Ulrich et al., 2016, 2017; Tordrup et al., 2025). This could potentially lead to an underestimation of the effects elicited by GIP. We also tested the activity of the co‐agonist on the rat GLP‐2 and GIP receptors. The GIP/GLP‐2 receptor co‐agonist (Co‐Ago) activated the rat GLP‐2 receptor as well as the GIP receptor with a lower potency compared to rat GLP‐2 (LogEC_50_ = −8.9 ± 0.1 and LogEC_50_ = −9.5 ± 0.1, respectively) (Figure 1a) and rat GIP (LogEC_50_ = −8.5 ± 0.1 and LogEC_50_ = −10.1 ± 0.1, respectively) (Figure 1b). Thus, the co‐agonist activated both rat receptors potently.

### 3.2 GLP‐2 and GIP acutely increase superior mesenteric artery blood flow in anesthetized rats

**FIGURE 2:**
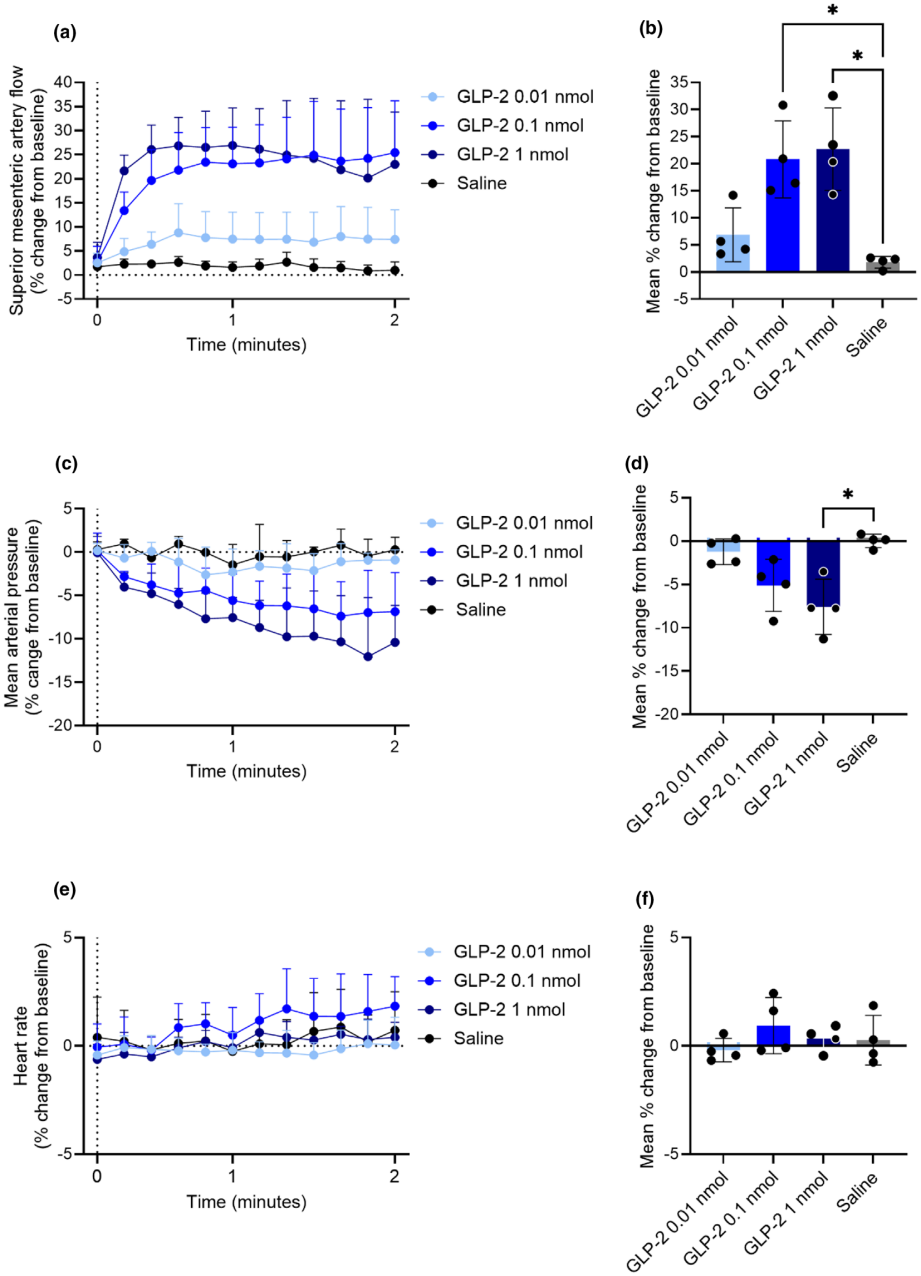
GLP‐2 acutely increases blood flow in the superior mesenteric artery. Percent change from baseline in (a and b) superior mesenteric artery blood flow, (c and d) mean arterial blood pressure, and (e and f) heart rate in response to an intravenous bolus injection of 0.01 nmol, 0.1 nmol, 1 nmol GLP‐2, and saline. *p* values by repeated measurements one‐way ANOVA, all compared to saline, corrected for multiple testing using the Dunnett test. Data shown as mean ± SD, *n* = 4.

In anesthetized rats, we tested the effect of three doses (0.01, 0.1, and 1 nmol) of GLP‐2 and GIP on superior mesenteric artery blood flow. The two highest doses of GLP‐2, 0.1 nmol and 1 nmol, increased superior mesenteric artery blood flow by 21 ± 7% and 23 ± 8%, respectively (both *p* = 0.03 when compared to saline), whereas 0.01 nmol GLP‐2 did not increase superior mesenteric artery blood flow compared to saline (*p* = 0.2) (Figure 2a,b). Likewise, 0.1 nmol and 1 nmol GLP‐2 decreased mean arterial blood pressure by −5% ± 3% and −8% ± 3%, respectively (*p* = 0.05 and *p* = 0.04, respectively, when compared to saline). In contrast, no effect of 0.01 nmol GLP‐2 was observed (*p* = 0.2 when compared to saline) (Figure 2c,d). GLP‐2 did not affect heart rate in any of the tested doses when compared to saline (1 nmol *p* = 0.9; 0.1 nmol *p* = 0.9; and 0.01 nmol *p* = 0.6) (Figure 2e,f). Thus, an acute response of GLP‐2, reaching a max effect at 0.1 nmol, was observed on superior mesenteric blood flow and mean arterial blood pressure. The actual values are shown in Figure S1.

**FIGURE 3:**
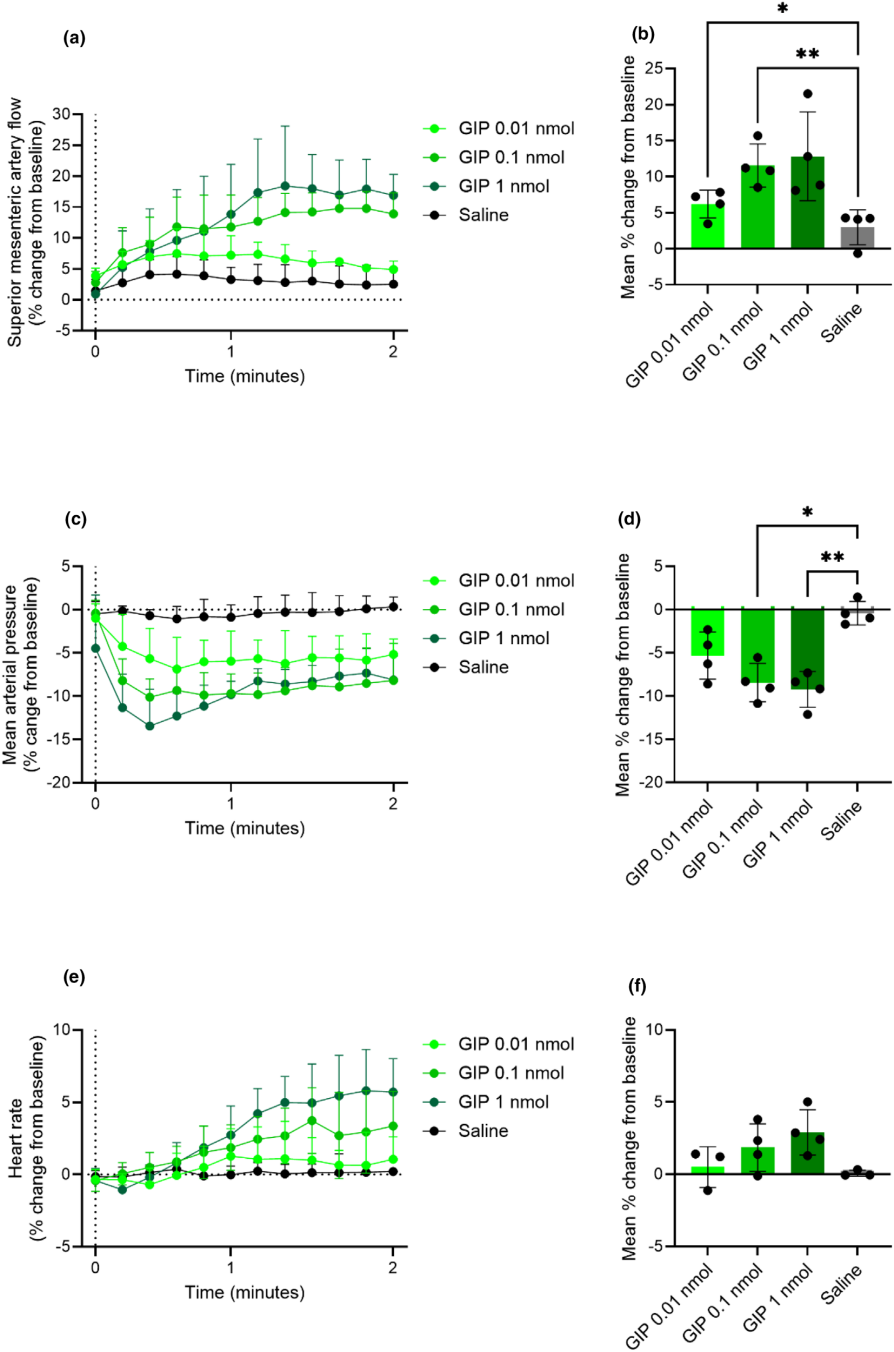
GIP acutely increases superior mesenteric artery blood flow. Percent change from baseline in (a and b) superior mesenteric artery blood flow, (c and d) mean arterial blood pressure, and (e and f) heart rate in response to an intravenous bolus injection of 0.01 nmol, 0.1 nmol, 1 nmol GIP, and saline. *p* Values by repeated measurements one‐way ANOVA, all compared to saline, corrected for multiple testing using the Dunnett test. Data shown as mean ± SD, *n* = 3–4.

GIP (0.01 nmol and 0.1 nmol) increased superior mesenteric artery blood flow by 6% ± 2% and 12% ± 3%, respectively (*p* = 0.01 and *p* = 0.009, respectively, when compared to saline) (Figure 3a,b). GIP at 0.1 nmol and 1 nmol decreased mean arterial blood pressure by −9% ± 3% and −9% ± 2%, respectively (*p* = 0.01 and *p* = 0.001, respectively, when compared to saline) (Figure 3c,d). Only the highest dose of GIP (1 nmol) tended to increase heart rate by 3% ± 0.8% (*p* = 0.2, when compared to saline) (Figure 3*E,F*). The actual values are shown in Figure S2.

We also investigated the effect of 1, 3, and 10 nmol GLP‐2 and GIP on superior mesenteric artery blood flow, mean arterial blood pressure, and heart rate. Within 2 min, 1, 3, and 10 nmol GLP‐2 increased superior mesenteric artery blood flow to the same degree, by 14% ± 6%, 11% ± 8%, and 11% ± 6%, respectively (*p* = 0.007, *p* = 0.07, and *p* = 0.04 respectively, when compared to saline). None of the three doses of GLP‐2 affected mean arterial blood pressure when compared to saline (1 nmol *p* = 0.2; 3 nmol *p* = 0.5; and 10 nmol *p* = 0.3) (Figure S3, and the actual values are shown in Figure S4). None of the tested doses of GIP consistently increased superior mesenteric artery blood flow when compared to saline (1 nmol *p* = 0.08; 3 nmol *p* = 0.8; and 10 nmol *p* = 0.6), nor did they consistently affect mean arterial blood pressure (1 nmol *p* = 0.3; 3 nmol *p* = 0.3; and 10 nmol *p* = 0.1), or heart rate (1 nmol *p* = 0.8; 3 nmol *p* = 0.7; and 10 nmol *p* = 0.9) when compared to saline (Figure S5, and the actual values are shown in Figure S6).

### 3.3 GLP‐2 and GIP do not act synergistically or additively to increase superior mesenteric artery blood flow in anesthetized rats

To test the hypothesis that GLP‐2 and GIP combined have a synergistic effect on superior mesenteric artery blood flow, we conducted a series of experiments in which GLP‐2 (0.1 nmol), GIP (0.1 nmol), GLP‐2 + GIP (both 0.05 nmol), and saline +1% BSA were given in a random order.

**FIGURE 4:**
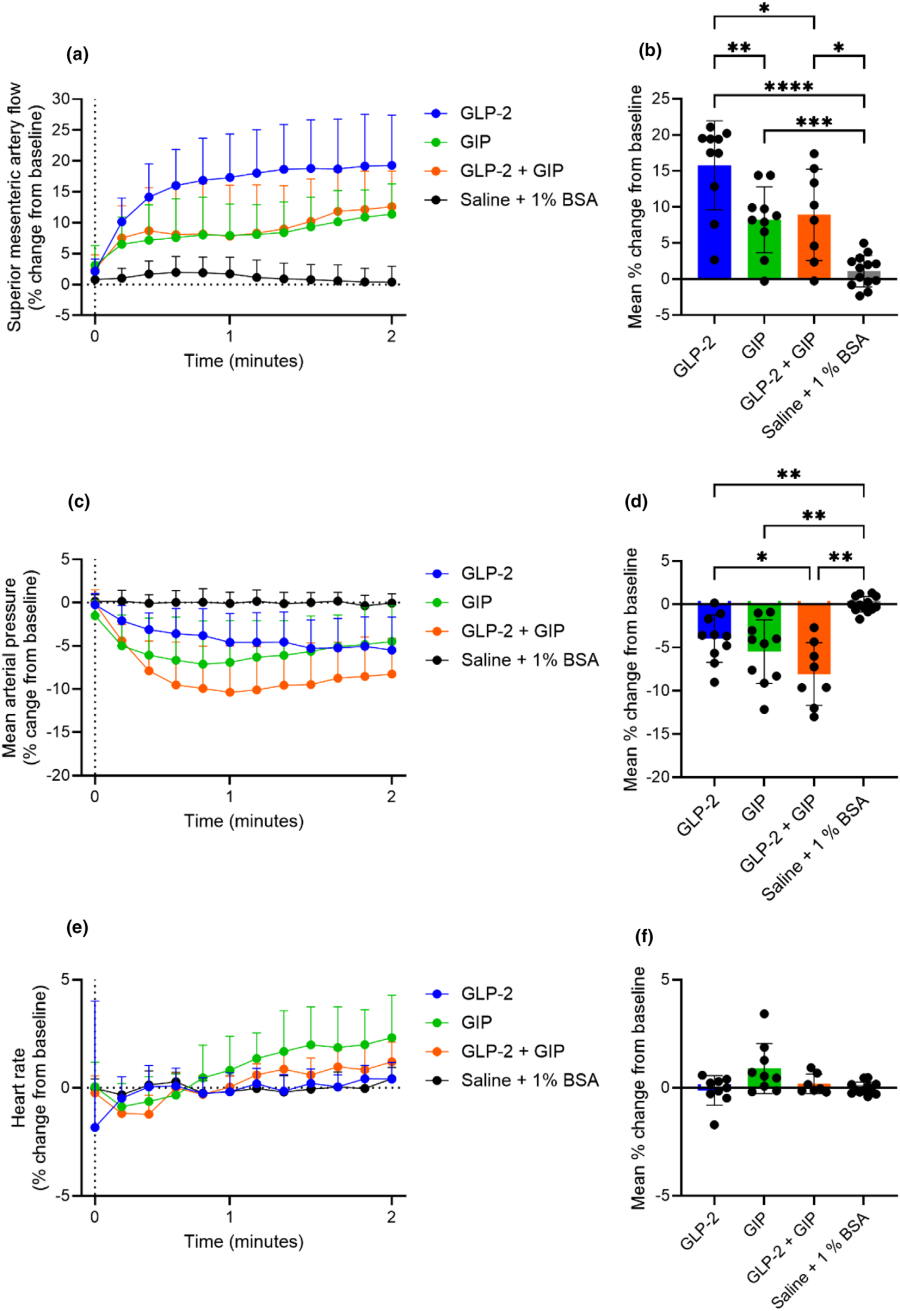
GLP‐2 and GIP do not act synergistically to increase superior mesenteric artery blood flow. Percent change in (a and b) **s**uperior mesenteric artery blood flow, (c and d) mean arterial blood pressure, and (e and f) heart rate in response to an intravenous bolus injection of 0.1 nmol GLP‐2, 0.1 nmol GIP, GLP‐2 + GIP both 0.05 nmol, and saline +1% BSA. *p* Values by repeated measurement mixed‐effects analysis, all groups compared, corrected for multiple testing using the Tukey test. Data shown as mean ± SD, *n* = 8–13.

In these experiments, GLP‐2 and GIP alone increased superior mesenteric artery blood flow when compared to saline (*p* < 0.0001 and *p* = 0.0003, respectively). GLP‐2 increased superior mesenteric artery blood flow to a larger extent than GIP, 16% ± 6% and 8% ± 5%, respectively (GLP‐2 vs. GIP; *p* < 0.007). GLP‐2 combined with GIP increased the superior mesenteric artery blood flow when compared to saline (*p* = 0.01); however, the response (9% ± 6% increase from baseline) was comparable to the response elicited by GIP (GLP‐2 + GIP vs. GIP; *p* = 0.9) and lower than that elicited by GLP‐2 (GLP‐2 + GIP vs. GLP‐2; *p* = 0.04) (Figure 4a,b). Thus, no synergistic response of GLP‐2 and GIP was observed. GLP‐2 and GIP alone decreased mean arterial blood pressure by −4% ± 3% and −6% ± 4%, respectively (*p* = 0.004 and *p* = 0.005, respectively, when compared to saline). The combination of GLP‐2 and GIP decreased the blood pressure by −8% ± 4%, (*p* = 0.002 when compared to saline), which was more than that elicited by GLP‐2 (GLP‐2 + GIP vs. GLP‐2; *p* = 0.02) (Figure 4*C,D*). Neither GLP‐2 alone nor GLP‐2 + GIP affected heart rate when compared to saline (*p* = 0.9 and *p* = 0.6, respectively); however, GIP alone tended to increase heart rate by 0.9% ± 0.4% (*p* = 0.2 when compared to saline (−0.2 ± 0.08%)) (Figure 4e,f). The actual values are shown in Figure S7.

To investigate whether an additive response could be elicited using lower doses of GLP‐2 and GIP, we conducted a separate series of experiments in which GLP‐2 (0.05 nmol), GIP (0.05 nmol), GLP‐2 + GIP (both 0.05 nmol), and saline +1% BSA were given in a random order. The responses were in general similar to those observed with the higher doses (Figure 4), and, thus, no additive effect of GLP‐2 and GIP on superior mesenteric blood flow was observed (Figure S8, and the actual values are shown in Figure S9).

The monomolecular GLP‐2/GIP receptor co‐agonist increased superior mesenteric blood flow by 22% ± 11% (*p* = 0.0006, when compared to saline) (Figure 5a,b). It decreased mean arterial blood pressure by −5% ± 2% (*p* = 0.008, when compared to saline) (Figure 5c,d) without affecting heart rate (*p* = 0.1, when compared to saline) (Figure 5e,f). The actual values are shown in Figure S10. Due to the experimental setup, the saline controls are the same in Figures S3, S5, and Figure 5, and the Figures S1–S18 showing the corresponding actual values (Figures S4, S6, and S10).

**FIGURE 5:**
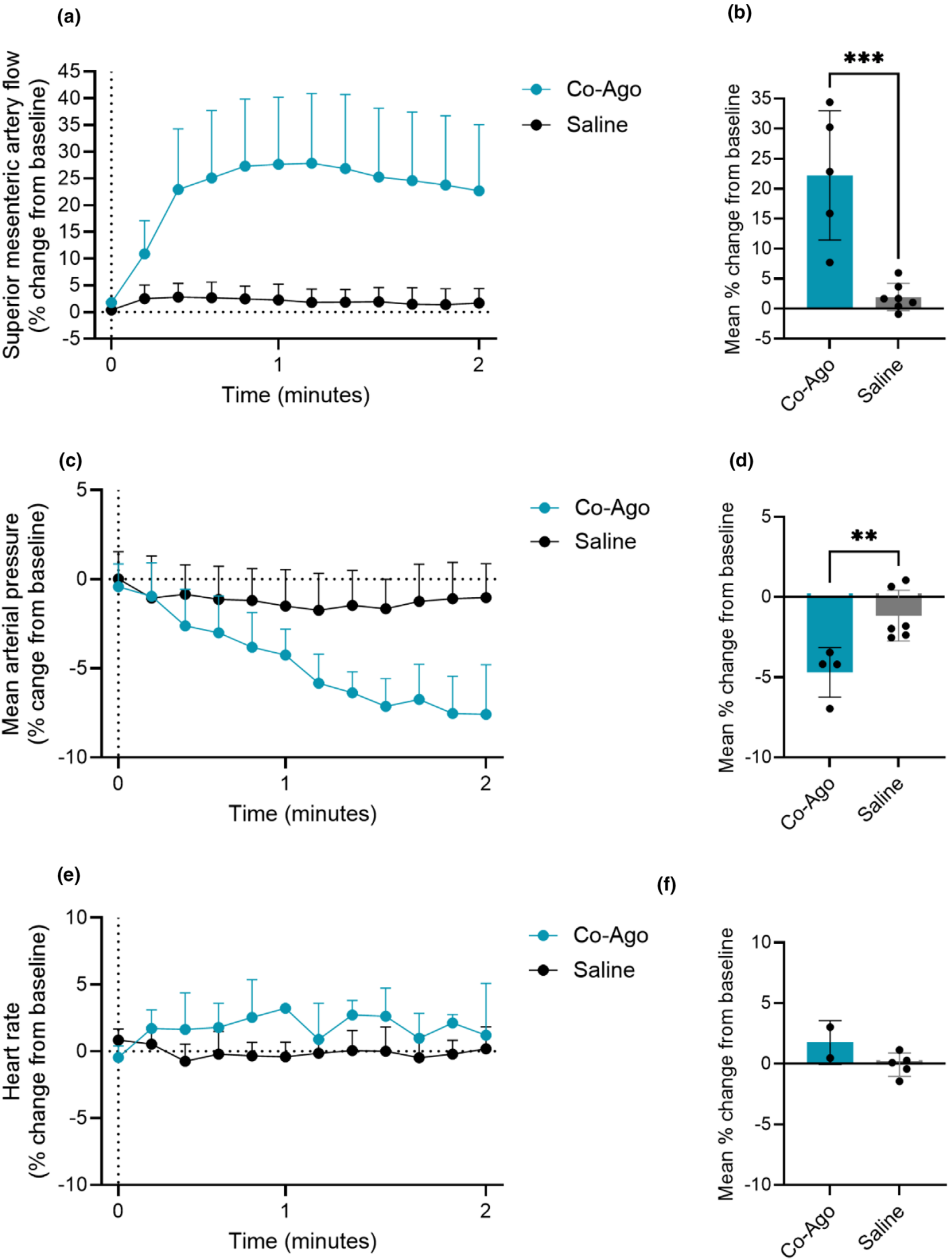
The GLP‐2/GIP receptor co‐agonist; Co‐Ago increases superior mesenteric artery blood flow in rats. Percent change from baseline in (a and b) superior mesenteric artery blood flow, (c and d) mean arterial blood pressure, and (e and f) heart rate in response to an intravenous bolus injection of 0.2 μmol Co‐Ago and saline. *p* Values by ordinary unpaired *t*‐test. Data shown as mean ± SD, *n* = 2–4. Due to the experimental setup, the saline controls are the same in Figures S3, S5, and Figure 5, and Figures S1–S18 showing the corresponding actual values (Figures S4, S6, and S10).

### 3.4 GLP‐2 and GIP increase superior mesenteric artery blood flow in anesthetized rats, independent of nitric oxide

To investigate whether the GLP‐2‐ and GIP‐induced increases in superior mesenteric artery blood flow are mediated by NO, we administered the NO synthase inhibitor L‐NAME via a continuous infusion i.v. 30 min prior to GLP‐2 or GIP stimulation. The study design is shown in Figure S11. When infused alone for 30 min, L‐NAME increased mean arterial blood pressure by 22.4 ± 4.1 mmHg, whereas superior mesenteric artery blood flow and heart rate were unaffected (Figures S12 and S13).

**FIGURE 6:**
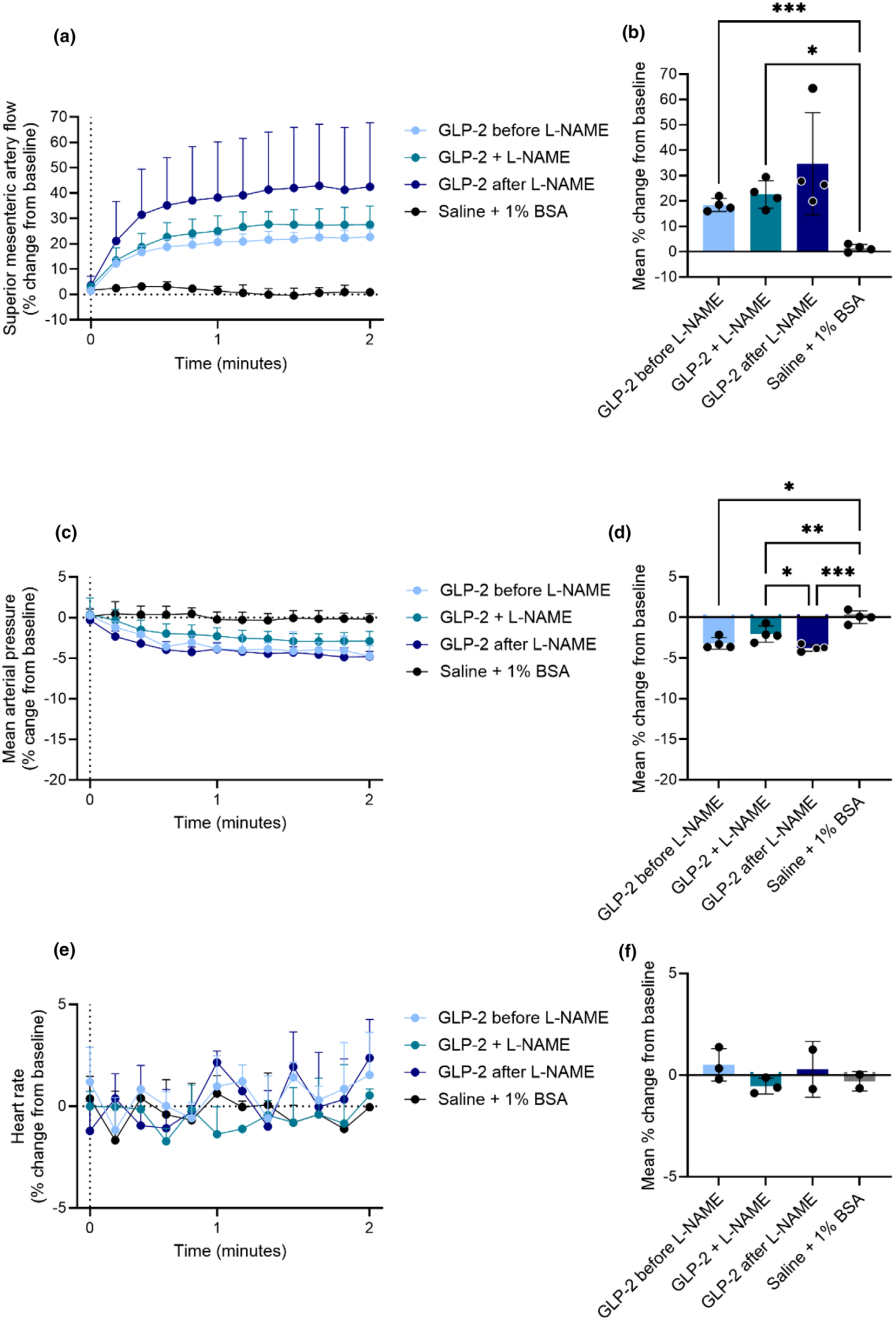
GLP‐2 increases superior mesenteric artery blood flow in rats independent of nitric oxide. Percent changes in (a and b) superior mesenteric artery blood flow, (c and d) mean arterial blood pressure, and (e and f) heart rate in response to an intravenous bolus injection of saline +1% BSA, or 0.1 nmol GLP‐2 before, during, and after an intravenous infusion of L‐NAME. *p* Values by repeated measurement one‐way ANOVA, except (f), which is by mixed‐effects analysis, all groups compared, corrected for multiple testing using the Tukey test. Data shown as mean ± SD, *n* = 2–4.

The increase in superior mesenteric artery blood flow elicited by 0.1 nmol GLP‐2 was the same before, during, and after the L‐NAME infusion (19% ± 3%, 23% ± 5%, and 35% ± 20%, respectively) (*p* > 0.4) (Figure 6a,b). The decrease in mean arterial blood pressure elicited by GLP‐2 was the same before, during, and after the L‐NAME infusion (−3% ± 1%, −2% ± 1%, and −4% ± 0.4%, respectively) (*p* > 0.03) (Figure 6*C,D*). GLP‐2 did not induce a change in heart rate before, during, or after the L‐NAME infusion (*p* > 0.2) (Figure 6e,f). The actual values are shown in Figure S12.

**FIGURE 7:**
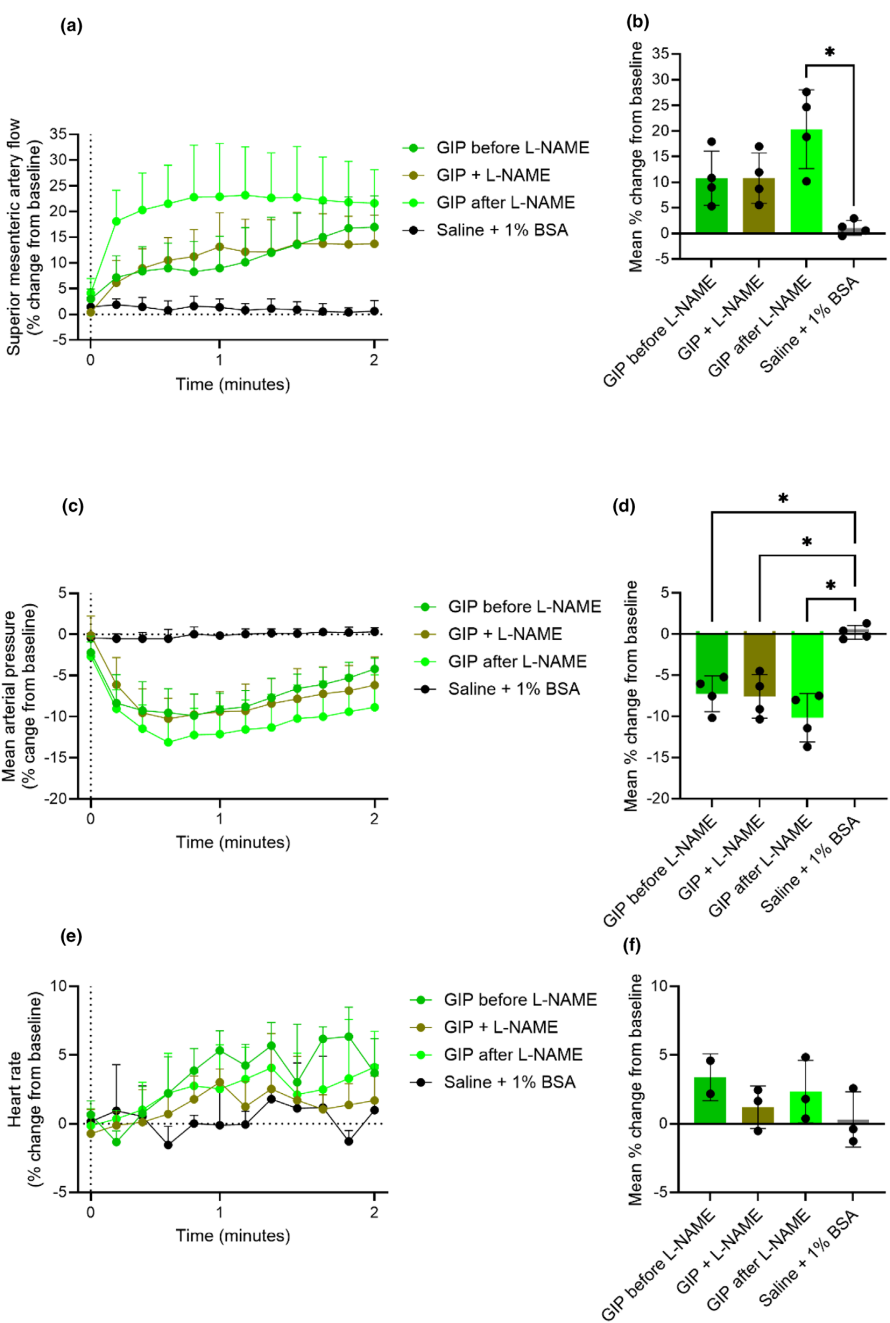
GIP increases superior mesenteric artery blood flow in rats independent of nitric oxide. Percent changes in (a and b) superior mesenteric artery blood flow, (c and d) mean arterial blood pressure, and (e and f) heart rate in response to an intravenous bolus injection of saline +1% BSA, or 0.1 nmol GIP before, during, and after an intravenous infusion of L‐NAME. *p* Values by repeated measurement one‐way ANOVA, except (f) which is by mixed‐effects analysis, all groups compared, corrected for multiple testing using the Tukey test. Data shown as mean ± SD, *n* = 2–4.

The increase in superior mesenteric artery blood flow elicited by 0.1 nmol GIP was the same before, during, and after the L‐NAME infusion (11% ± 5%, 11% ± 5%, and 20% ± 8%, respectively) (*p* > 0.1) (Figure 7a,b), however, the response following the L‐NAME infusion surprisingly seemed to be higher than the two previous responses, a tendency also observed with GLP‐2 stimulation. The decrease in mean arterial blood pressure elicited by GIP was the same before, during, and after the L‐NAME infusion (−7% ± 2%, −8% ± 3%, and −10% ± 3%, respectively) (*p* > 0.1) (Figure 7c,d). GIP did not significantly change the heart rate before, during, or after the L‐NAME infusion (*p* > 0.2), although the heart rate tended to increase in response to GIP (Figure 7e,f), which is consistent with the results shown in Figures 3 and 4. The actual values are shown in Figure S13.

### 3.5 GLP‐2 and GIP increase superior mesenteric artery blood flow in anesthetized rats independently of vasoactive intestinal polypeptide

To investigate whether GLP‐2 and GIP increase blood flow in the superior mesenteric artery indirectly via VIP, we administered a VIP receptor antagonist (VIPR‐An) (Study design shown in Figure S14). When infused alone for 30 min, the VIPR‐An did not affect basal superior mesenteric artery blood flow, mean arterial blood pressure, or heart rate (Figures S15 and S16).

**FIGURE 8:**
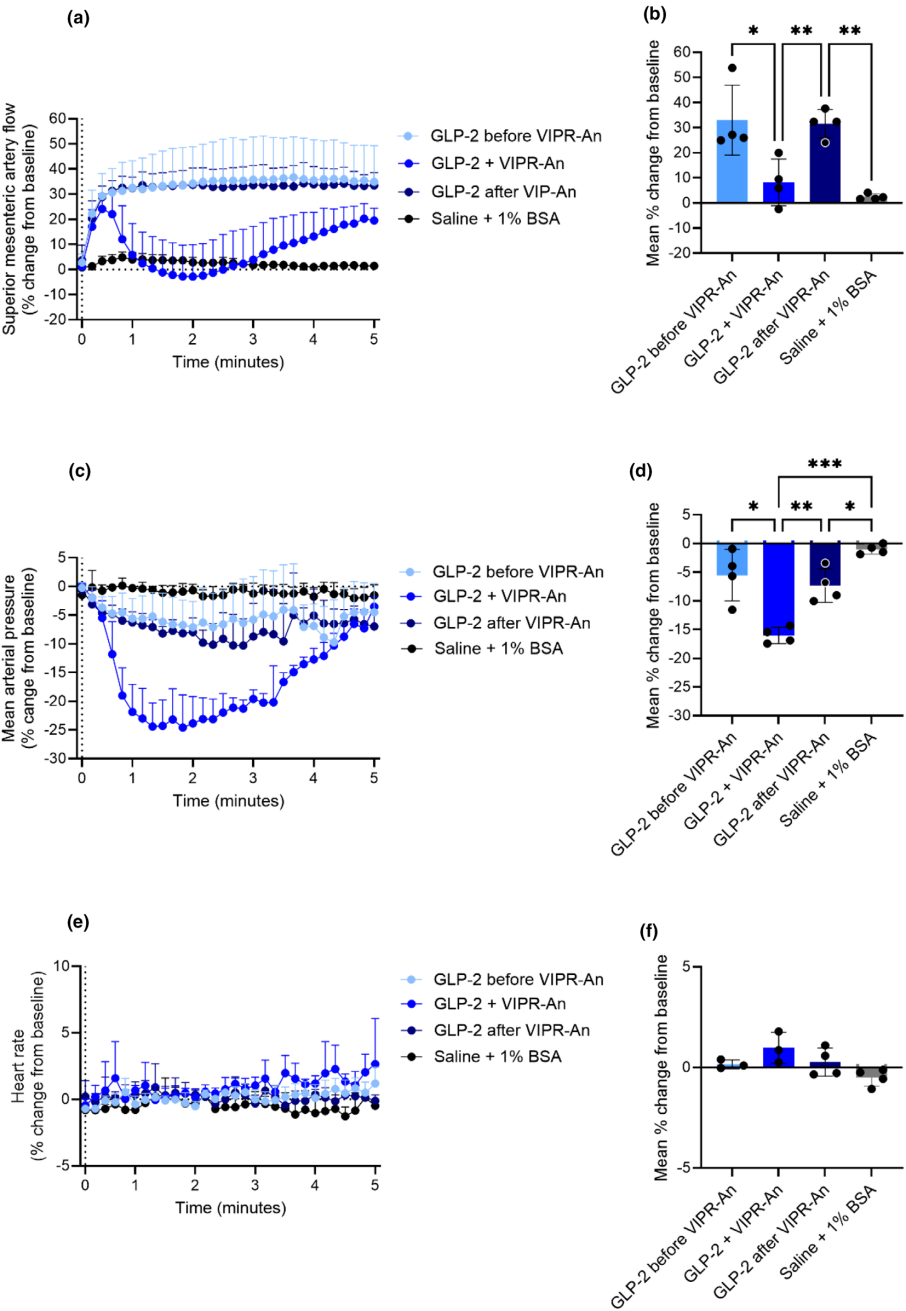
GLP‐2 increases superior mesenteric artery blood flow in rats independent of VIP. Percent changes in (a and b) superior mesenteric artery blood flow, (c and d) mean arterial blood pressure, and (e and f) heart rate in response to an intravenous bolus injection of saline +1% BSA or 0.1 nmol GLP‐2 before, during, and after an intravenous infusion of a VIP receptor antagonist (VIPR‐An). *p* Values by repeated measurement one‐way ANOVA, except (f), which is by mixed‐effects analysis, all groups compared, corrected for multiple testing using the Tukey test. Data shown as mean ± SD, *n* = 3–4.

GLP‐2 (0.1 nmol) increased superior mesenteric artery blood flow to the same degree before and after VIPR‐An infusion (33% ± 14% and 32% ± 3%, respectively) (*p* = 0.9). However, during VIPR‐An infusion, the 0.1 nmol GLP‐2 bolus elicited a quick initial increase in superior mesenteric artery blood flow followed by a rapid decrease. Hereafter, blood flow slowly increased to levels similar to those observed in the other GLP‐2 stimulations. Thus, there was no significant increase in superior mesenteric artery blood flow during the initial 5 min compared to saline during VIPR‐An infusion (*p* = 0.6) (Figure 8a,b). Looking at changes during GLP‐2 and VIPR‐An co‐infusion, we found that vascular resistance rapidly decreased and stayed low throughout the infusion. This strongly suggests that the greater decrease in mean arterial blood pressure induced by GLP‐2 during the VIPR‐An infusion was responsible for the observed SMA flow decrease. The decrease in mean arterial blood pressure caused by the co‐infusion of GLP‐2 and VIPR‐An was significantly larger compared to the decrease induced by GLP‐2 before and after VIPR‐An infusion (*p* = 0.03 and *p* = 0.006, respectively) (Figure 8c,d). Even so, GLP‐2 did not alter the heart rate before, during, or after the VIPR‐An infusion compared to saline (*p* > 0.3) (Figure 8e,f). The actual values are shown in Figure S15.

**FIGURE 9:**
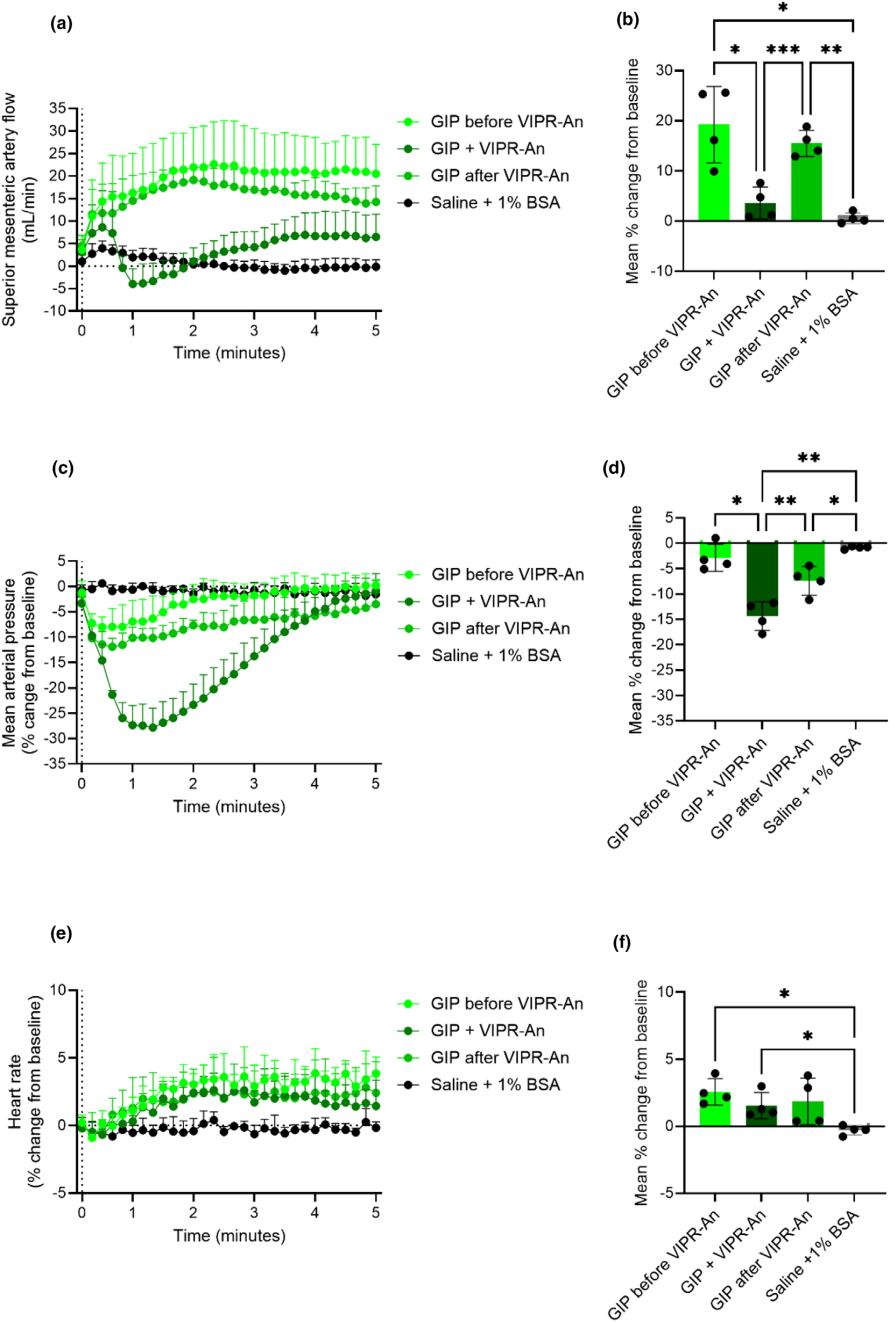
GIP increases superior mesenteric artery blood flow in rats independent of VIP. Percent changes in (a and b) superior mesenteric artery blood flow, (c and d) mean arterial blood pressure, and (e and f) heart rate in response to an intravenous bolus injection of saline +1% BSA, or 0.1 nmol GIP before, during, and after an intravenous infusion of a VIP receptor antagonist (VIPR‐An). *p* Values by repeated measurement one‐way ANOVA, all groups compared, corrected for multiple testing using the Tukey test. Data shown as mean ± SD, *n* = 4.

When investigating whether the effects of GIP were dependent on VIP, we observed a pattern similar to that observed with GLP‐2 (Figure 9). In contrast to GLP‐2, GIP increased heart rate before and during the VIPR‐An infusion compared to saline (*p* = 0.02 and *p* = 0.04, respectively). After the VIPR‐An infusion, GIP did not affect heart rate (*p* = 0.2, when compared to saline) (Figure 9e,f). The actual values are shown in Figure S16.

### 3.6 GLP‐2(3–33) reduces the GLP‐2‐induced increase in superior mesenteric artery blood flow in anesthetized rats

The truncated GLP‐2 metabolite, GLP‐2(3–33), is an antagonist of the GLP‐2 receptor (Gabe, Gasbjerg, et al., 2022; Gadgaard et al., 2022), and we here investigated its inhibition of the GLP‐2‐induced increase in superior mesenteric artery blood flow (Study design in Figure S17). When infused alone for 30 min, GLP‐2(3–33) did not affect basal superior mesenteric artery blood flow, mean arterial blood pressure, or heart rate (Figure S18).

**FIGURE 10:**
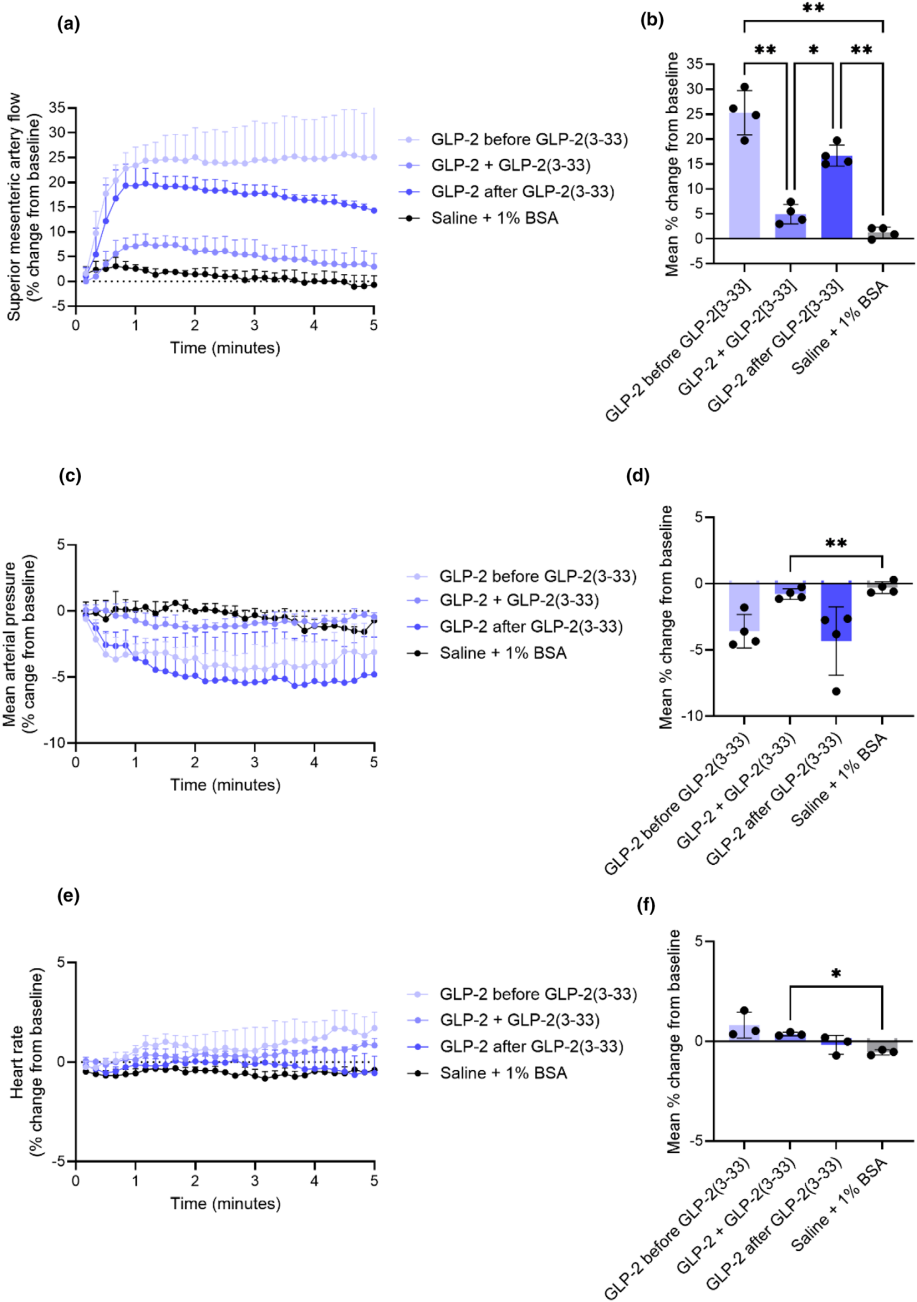
GLP‐2(3–33) reduces the GLP‐2‐induced increase in superior mesenteric artery blood flow in rats. Percent changes in (a and b) superior mesenteric artery blood flow, (c and d) mean arterial blood pressure, and (e and f) heart rate in response to an intravenous bolus injection of saline +1% BSA, or 0.05 nmol GLP‐2 before, during, and after an intravenous infusion of GLP‐2(3–33). *p* Values by repeated measurement one‐way ANOVA, all groups compared, corrected for multiple testing using the Tukey test. Data shown as mean ± SD, *n* = 3–4.

The increase in superior mesenteric artery blood flow elicited by GLP‐2 (0.05 nmol) was not different before and after the GLP‐2(3–33) infusion (25.3% ± 2.2% and 16.7% ± 0.5%, respectively) (*p* = 0.09). When given during GLP‐2(3–33) infusion, the blood flow increase elicited by GLP‐2 (4.9% ± 1.0%) was markedly reduced compared to the response obtained before or after GLP‐2(3–33) infusion (*p* = 0.002 and *p* = 0.01, respectively) (Figure 10a,b). The decrease in blood pressure induced by GLP‐2 during the GLP‐2(3–33) infusion was smaller than the decrease induced by GLP‐2 before and after GLP‐2(3–33) infusion, but the difference was not significant (*p* = 0.07 and *p* = 0.2, respectively) (Figure 10c,d). The effect of GLP‐2 on heart rate was not different before, during, or after the GLP‐2(3–33) infusion (*p* > 0.1) (Figure 10e,f). The actual values are shown in Figure S18.

## 4 DISCUSSION

In anesthetized rats, we found that GLP‐2 acutely increased superior mesenteric artery blood flow and decreased mean arterial blood pressure with no effect on heart rate. Similarly, GIP acutely increased superior mesenteric artery blood flow, decreased mean arterial blood pressure, and, in contrast to GLP‐2, increased heart rate slightly. A GLP‐2/GIP receptor co‐agonist had effects comparable to the native hormones as it increased superior mesenteric artery blood flow and decreased mean arterial blood pressure. We found no additive or synergistic effects of GLP‐2 and GIP, and the vasodilatory effects were independent of NO and VIP. Finally, we found that GLP‐2(3–33) drastically diminished the GLP‐2‐induced increase in superior mesenteric artery blood flow.

Our observations are consistent with reported increases in superior mesenteric artery blood flow after GLP‐2 administration in healthy humans, individuals with short bowel syndrome, calves, and piglets (Bremholm et al., 2009, 2011; Guan et al., 2003; Høyerup et al., 2013; Taylor‐Edwards et al., 2011). In a study also using the ultrasonic transit time technique, GLP‐2 infusion of doses similar to and higher than those we administered (ranging from 0.9 nmol/kg to 93 nmol/kg) dose‐dependently increased superior mesenteric artery blood flow in anesthetized rats (Deniz et al., 2007). The blood flow in the celiac, renal, and left gastric arteries did not respond to GLP‐2, whereas the flow in the carotid and inferior mesenteric arteries gradually increased after 30 min of GLP‐2 infusion. However, the increase was consistently larger in the superior mesenteric artery (Deniz et al., 2007). In contrast to our findings, GLP‐2 did not cause any significant change in mean arterial blood pressure (Deniz et al., 2007).

In line with our observations, GIP increased superior mesenteric artery and portal vein blood flow (Rasmussen et al., 2025), and jejunal and duodenal blood flow in humans (Honka et al., 2017; Koffert et al., 2017). GIP infusion also increased small intestinal blood flow (and decreased pancreatic blood flow) in anesthetized dogs (Kogire et al., 1988) and cats, but in contrast to our results, arterial pressure remained unchanged (Fara & Salazar, 1978). In dogs, GIP had no effect on femoral artery blood flow (Fara & Salazar, 1978) and celiac artery flow (Kogire et al., 1988), whereas in humans, GIP infusion increased femoral artery blood flow during hyperglycemic pancreatic clamp conditions, but no effect was observed during basal or euglycemic conditions, and neither was an effect of GIP observed on carotid or brachial artery blood flow (Karstoft et al., 2015). Conflicting studies report that GIP decreased duodenal blood flow in normoglycemic rats (Svensson et al., 1997), and that infusions of different doses of GIP, the highest dose (0.01 nmol/kg × min), decreased blood perfusion of the whole pancreas, pancreatic islets, duodenum, colon, liver, and kidneys (measured using microsphere technique) in anesthetized rats (Gao et al., 2018).

We found both GLP‐2 and GIP to be potent stimulators of superior mesenteric artery blood flow; therefore, we tested the hypothesis that GLP‐2 and GIP, when combined, exert a synergistic stimulatory effect. This hypothesis was not supported. Surprisingly, we found that GLP‐2 had the largest effect on superior mesenteric artery blood flow, and that the effect of GLP‐2 + GIP was comparable to that of GIP. This may be due to GLP‐2 + GIP resulting in a greater decrease in mean arterial blood pressure than GLP‐2 alone, thereby limiting the increase in superior mesenteric blood flow, which is generally associated with lowered blood pressure. To further explore the combination of GLP‐2 and GIP, we tested a GLP‐2/GIP receptor co‐agonist. The co‐agonist activated both the rat GLP‐2 and GIP receptors, although with lower potencies than the native hormones (Sparre‐Ulrich et al., 2016, 2017; Tordrup et al., 2025). The co‐agonist increased superior mesenteric blood flow while causing a decrease in mean arterial blood pressure, thus having effects comparable with the native hormones. However, the co‐agonist was given at a much higher dose than the native hormones, thus making a direct comparison challenging. It is, however, likely that we observed the maximum effect of the co‐agonist and that these effects could perhaps also have been observed at lower doses, since we observed that the native hormones gave an almost equal response at 1 and 0.1 nmol.

GLP‐2 receptors co‐localized with eNOS‐containing submucosal neurons in the GI tract of pigs and humans (Guan et al., 2006) and GLP‐2 is reported to increase eNOS activity and protein abundance in pigs (Guan et al., 2003). In line with this, the GLP‐2‐mediated increase in blood flow is reduced but not eliminated by L‐NAME in both pigs and rats (Deniz et al., 2007; Guan et al., 2003; Gulec Suyen et al., 2015), thus questioning the dependency of NO. GIP stimulates NO production from portal vein endothelial cells (but not from hepatic artery endothelial cells) (Ding et al., 2004). We therefore investigated whether GLP‐2 and GIP stimulated superior mesenteric blood flow via NO. We found that both GLP‐2 and GIP increased superior mesenteric artery blood flow independently of NO, based on the lack of effect of L‐NAME (at a concentration high enough to cause an increase in mean arterial blood pressure, attesting to its effectiveness).

GLP‐2 receptors also co‐localize with enteric neurons containing VIP in the GI tract (Amato et al., 2010; Cinci et al., 2011; Guan et al., 2006). VIP is a peptide neurotransmitter and a relaxant of circular smooth muscle of the gastric fundus and the entire intestine (Bojö et al., 1993; Grider et al., 1985), and GLP‐2 treatment increases the number of VIP‐expressing neurons (de Heuvel et al., 2012; Sigalet et al., 2007, 2010). Furthermore, GLP‐2‐induced gastric relaxation, gastric motility (Amato et al., 2009; Traini et al., 2020), and anti‐inflammatory processes (Sigalet et al., 2007) are mediated by VIP released from enteric neurons. In contrast, neither L‐NAME nor a VIPR‐An was able to abolish a GLP‐2‐induced inhibitory effect on the motility of isolated murine proximal colon (Amato et al., 2010). We found that GLP‐2 and GIP can initially increase mesenteric blood flow during VIPR‐An infusion. Therefore, we suggest that the flow‐mediating effects are independent of VIP. However, due to a significant reduction in mean arterial pressure (Figure 8c,d and Figure 9c,d), the increased blood flow during VIPR‐An infusion (Figure 8a,b and Figure 9a,b) was not maintained throughout the infusion period, thus making it difficult to draw firm conclusions. The reduction in blood pressure could be due to partial agonism by the VIPR‐An but could also suggest perturbations of other components involved in vasodilation, such as the baroreflex machinery which regulates blood pressure (Wehrwein & Joyner, 2013). Specifically, the rapid and significant reduction in arterial blood pressure elicited during GLP‐2 + VIPR‐An co‐infusion did not induce a rise in heart rate as would have been expected as a consequence of the reduction in blood pressure. Further investigations are needed to address this phenomenon and the potential involvement of VIP.

The GIP receptor is present in endothelial cells (Zhong et al., 2000) but possibly also in smooth muscles cells (Berglund et al., 2015), pointing to a possible direct effect of GIP on the vasculature, and this is supported by our observations that GIP increases superior mesenteric blood flow independently of NO and VIP. In contrast, in the GI tract, the GLP‐2 receptor has been found in villus epithelium (primarily in the jejunum with a predominance in the luminal part of the villi (Thulesen et al., 2000)), myenteric plexus, and mucosal endocrine cells (Guan et al., 2006; Yusta et al., 2000), on subepithelial myofibroblasts (Gadgaard et al., 2022; Ørskov et al., 2005), and on enteric neurons in the muscularis layer (Bjerknes & Cheng, 2001; Guan et al., 2006), as well as enteric neurons, but not in the epithelium of rat jejunal mucosa (Pedersen et al., 2015). Thus, the localization of the GLP‐2 receptor reported so far does not support a direct effect of GLP‐2 on the vasculature. However, GLP‐2(3–33), which is a weak partial agonist with antagonistic properties on the GLP‐2 receptor (Thulesen et al., 2002), strongly diminished the GLP‐2‐induced increase in superior mesenteric artery blood flow which could suggest that the effect was transmitted via the GLP‐2 receptor. Another possibility is that GLP‐2 act through another receptor to increase superior mesenteric artery blood flow. Supporting this GLP‐2 activates the GIP receptor (Skov‐Jeppesen et al., 2019), and the GLP‐1 receptor, an effect that is abolished by GLP‐2(3–33) (Gabe, Gasbjerg, et al., 2022; Gadgaard et al., 2022). The latter might be a less likely mechanism as neither GLP‐1, GLP‐1_9–36 amide_, nor exenatide had an effect on blood flow in the mesenteric or renal arteries in humans (Bremholm et al., 2017).

A limitation to our study is that we used human GIP, which is a comparatively weak agonist on the rat GIP receptor with an E_max_ of 75% and no differences in binding affinities (Sparre‐Ulrich et al., 2016, 2017). This may have resulted in an underestimation of the effect of GIP; however, 0.1 and 1 nmol GIP resulted in nearly identical responses, suggesting that a maximal effect of GIP was reached at 0.1 nmol in our setup. Human GLP‐2, on the other hand, has similar activation profiles on the rat and human GLP‐2 receptor (Gadgaard et al., 2023). Additionally, we investigated the effects of bolus injections and their acute effects (2–5 min); thus, we cannot make conclusions regarding more long‐term or chronic effects. Similarly, the L‐NAME and VIPR‐An were only infused 30 min prior to stimulation; thus, prohibiting conclusions regarding long‐term effects. In addition to this, the VIPR‐An is not well described and might show agonistic properties. Technical issues prevented the collection of heart rate data in some experiments, thereby limiting the reliability of these data. We only studied anesthetized male rats; thus, limiting our results to this particular sex and species. Additionally, the rats were not fasted and had unlimited access to food; thus, the individual food intake of the rat might have affected our results, as splanchnic blood flow increases after a meal. Finally, the infusion of GLP‐2(3–33) was stopped 40 min before the last bolus of GLP‐2 was given. The half‐life of GLP‐2(3–33) is 27 min in humans, whereas the half‐life of GLP‐2(1–33) is 7 min (Hartmann, 2000), the half‐life is presumably four times lower in rats (Caldwell et al., 2004), however, some GLP‐2(3–33) might still be present during the final period of the experiment, which is in line with the tendency to a reduced rise in superior mesenteric blood flow after the GLP‐2(3–33) infusion compared to the first response. We did not perform any power calculations before conducting the studies; thus, there is a risk of type 2 errors considering the limited number of animals included in each experiment. However, we believe that, collectively, the experiments clearly demonstrate the effects of the peptides, and the robust responses observed across most experiments support the generalizability of the results.

Our results establish GLP‐2 and GIP as potent stimulators of superior mesenteric artery blood flow. This is important as GLP‐2 is currently considered in the treatment of short bowel syndrome induced intestinal failure (Jeppesen et al., 1999, 2001, 2005, 2009, 2011), Crohn's disease (Buchman et al., 2010), inflammatory bowel disease (Alavi et al., 2000; Xiao et al., 2000), chemotherapeutic enteritis (Tavakkolizadeh et al., 2000), intestinal ischemia (Guan et al., 2005; Moreira et al., 2024; Rajeevprasad et al., 2000), and in combination with GLP‐1, also obesity (Pálsson et al., 2024). The increased blood flow could be an important mediator of the beneficial effects of GLP‐2 in the abovementioned indications. Furthermore, the specific effect of GIP on intestinal parameters has been overshadowed by its insulinotropic effects, and it is important to consider the potent effect of not only GIP but also GIP receptor antagonists on splanchnic blood flow during the implementation of GLP‐1/GIP receptor co‐agonists in the treatment of metabolic diseases (Jastreboff et al., 2022; Rosenstock et al., 2021).

In conclusion, we found that GLP‐2, GIP, and a GLP‐2/GIP receptor co‐agonist acutely increased superior mesenteric artery blood flow and decreased mean arterial blood pressure in anesthetized rats. We found no additive or synergistic effects of GLP‐2 and GIP and found the vasodilatory effects to be independent of NO and VIP. Finally, we found that GLP‐2(3–33) could antagonize the vasodilatory effects of GLP‐2.

## AUTHOR CONTRIBUTIONS

K.D.G., B.H., J.J.H., L.S.G., and C.M.S. were involved in conceptualization. K.D.G. was involved in data curation. K.D.G., M.M.R., and C.M.S were involved in formal analysis. K.D.G., L.S.G., and C.M.S. were involved in funding acquisition. B.H., J.J.H., L.S.G., and C.M.S. were involved in project administration/supervision. K.D.G. was involved in writing—original draft. B.H., M.M.R., J.J.H., L.S.G., and C.M.S. were involved in writing—review and editing. All authors revised and approved the final version of the manuscript.

## FUNDING INFORMATION

This study is supported by the BRIDGE‐Translational Excellence Programme (bridge.ku.dk) at the Faculty of Health and Medical Sciences, University of Copenhagen, Copenhagen, funded by the Novo Nordisk Foundation, Grant agreement no. NNF20SA0064340 and by a collaboratory grant from the Department of Biomedical Sciences, University of Copenhagen. J.J.H. is supported by the Novo Nordisk Foundation (NNF) Center for Basic Metabolic Research, University of Copenhagen (NNF Application Number: 13563). Novo Nordisk Foundation Center for Basic Metabolic Research is an independent Research Center, based at the University of Copenhagen, Denmark and partially funded by an unconditional donation from the Novo Nordisk Foundation (Grant number NNF18CC0034900 and NNF23SA0084103).

## CONFLICT OF INTEREST STATEMENT

M.M.R., J.J.H., and L.S.G. are minority shareholders of Antag Therapeutics.

## ETHICS STATEMENT

All animal studies were approved by the Danish Animal Experiments Inspectorate (2020‐15‐0201‐00547) and the local ethical committee (Department of Experimental Medicine).

## Supporting information


Figures S1–S18.

## Data Availability

The actual values for all the following experiments, except the in vitro experiments, are shown in the Figures S1–S18.
