# Supplementary material for: GLP‐2 and GIP acutely increase superior mesenteric artery blood flow in male rats, and the effect is independent of nitric oxide and vasoactive intestinal peptide
Source: Physiol Rep. 2025 Dec 13;13(23):e70699. doi: 10.14814/phy2.70699 (PMC12701523; doi:10.14814/phy2.70699)
Supplement: Supplementary file 1 — Figures S1–S18. [file PHY2-13-e70699-s001.docx]

# Supplementary material


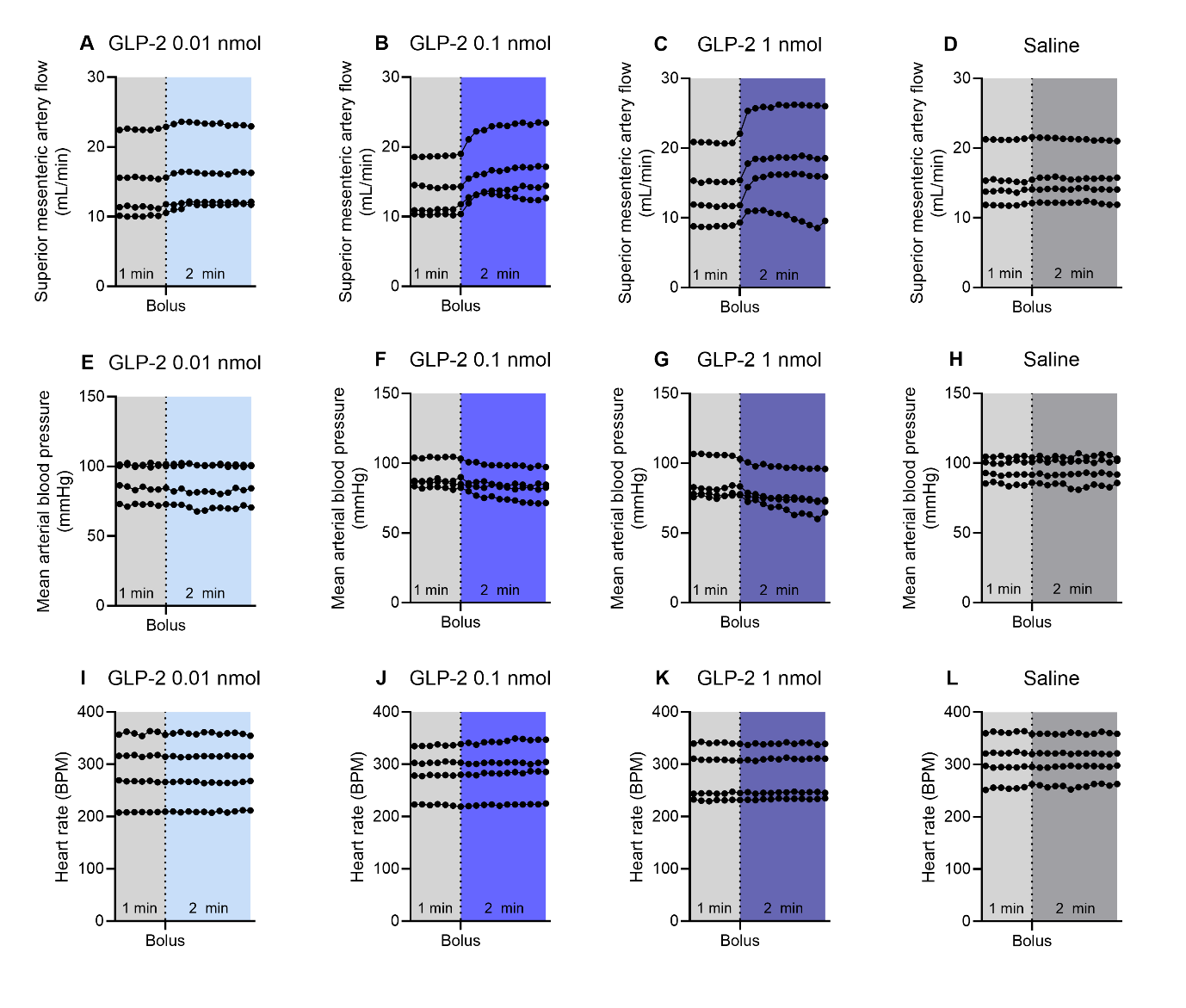
 **Supplementary Figure 1. GLP-2 acutely increases superior mesenteric blood flow.** (*A*-*D*) Superior mesenteric artery blood flow, (*E*-*H*) mean arterial blood pressure, and (*I*-*L*) heart rate in response to an intravenous bolus injection of (*A, E, I*) 0.01 nmol, (*B, F, J*) 0.1 nmol, (*C, G, K*) 1 nmol GLP-2, and (*D, H, L*) saline.


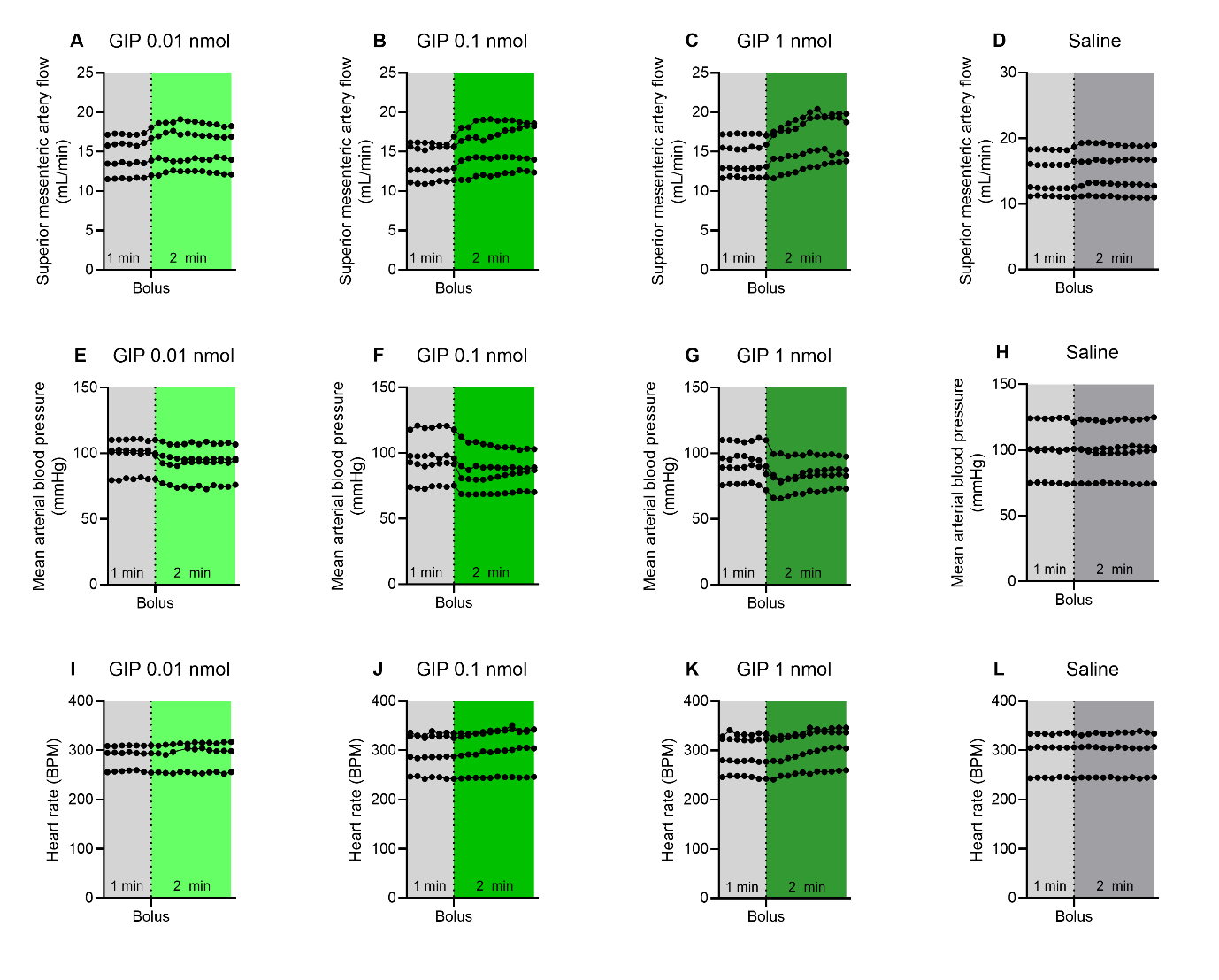


**Supplementary Figure 2. GIP acutely increases superior mesenteric blood flow.** (*A*-*D*) Superior mesenteric artery blood flow, (*E*-*H*) mean arterial blood pressure, and (*I*-*L*) heart rate in response to an intravenous bolus injection of (*A, E, I*) 0.01 nmol, (*B, F, J*) 0.1 nmol, (*C, G, K*) 1 nmol GIP, and (*D, H, L*) saline.

#
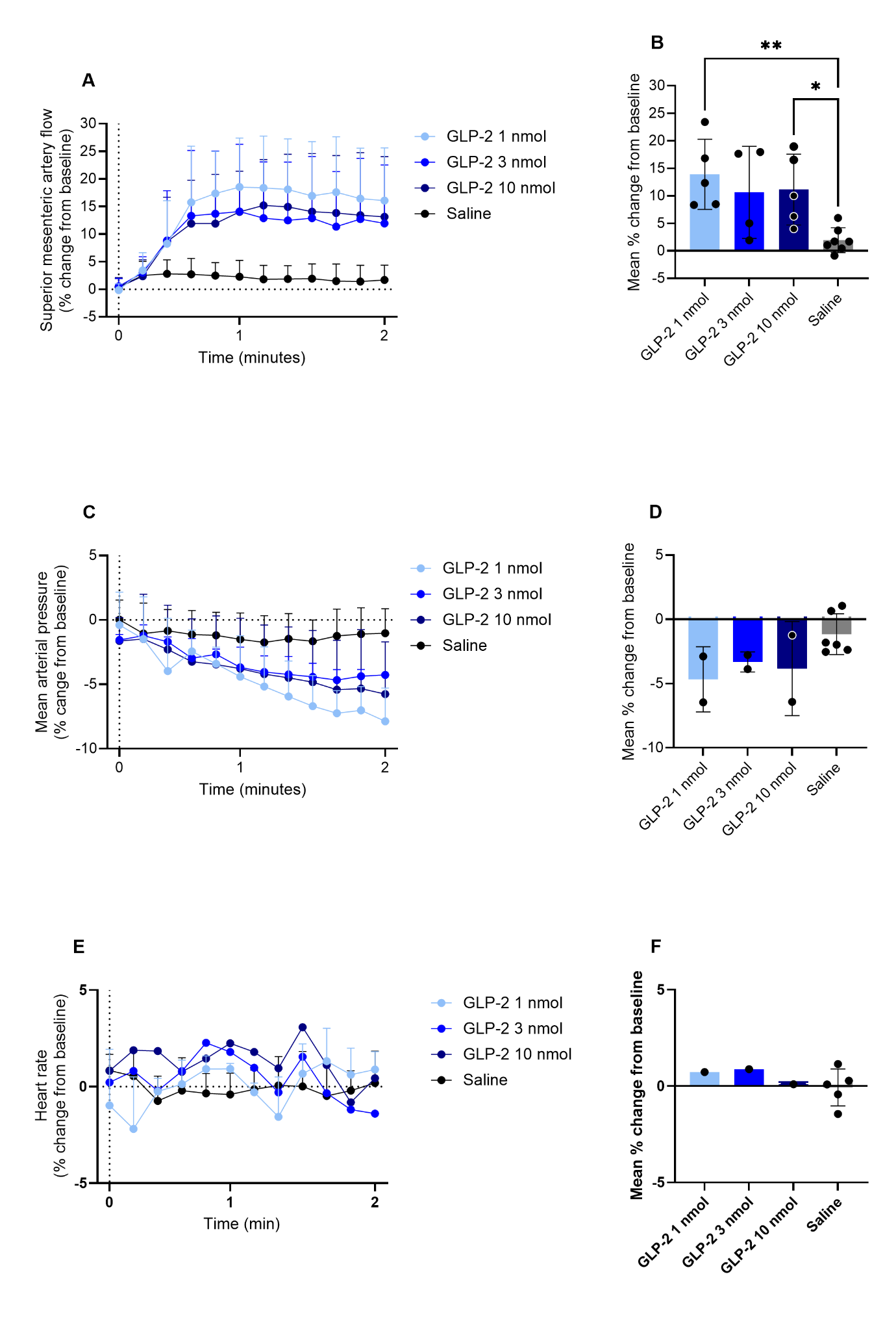


**Supplementary Figure 3**. **GLP-2 increases superior mesenteric artery flow in rats**. Percent change from baseline in (*A* and *B*) superior mesenteric artery blood flow and (*C* and *D*) mean arterial blood pressure in response to an intravenous bolus injection of 1 nmol, 3 nmol, 10 nmol GLP-2, and saline. P-values by ordinary one-way ANOVA, all compared to saline, corrected for multiple testing using the Dunnett test**.** Data shown as mean±SD, n=2-5. Due to the experimental setup the saline controls are the same in Supplementary Figure 3, 5, and Figure 5, and the Supplementary Figures showing the corresponding actual values (Supplementary Figure 4, 6, and 10).


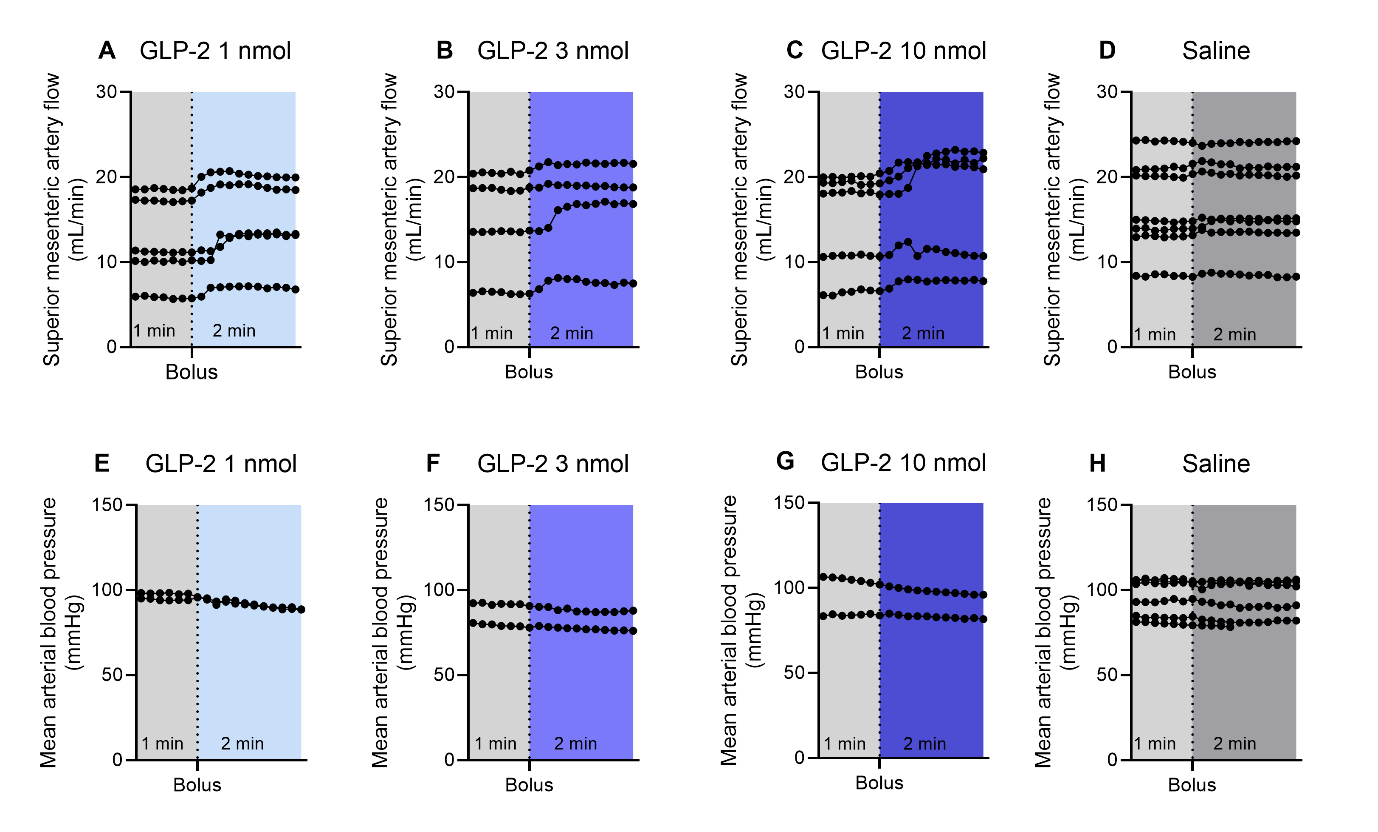


**Supplementary Figure 4**. **GLP-2 increases superior mesenteric artery flow in rats**. (*A*-*D*) Superior mesenteric artery blood flow and (*E*-*H*) mean arterial blood pressure in response to an intravenous bolus injection of (*A* and *E*) 1 nmol, (*B* and *F*) 3 nmol, (*C* and *G*) 10 nmol GLP-2, and (*D* and *H*) saline. Due to the experimental setup the saline controls are the same in Supplementary Figure 3, 5, and Figure 5, and the Supplementary Figures showing the corresponding actual values (Supplementary Figure 4, 6, and 10).


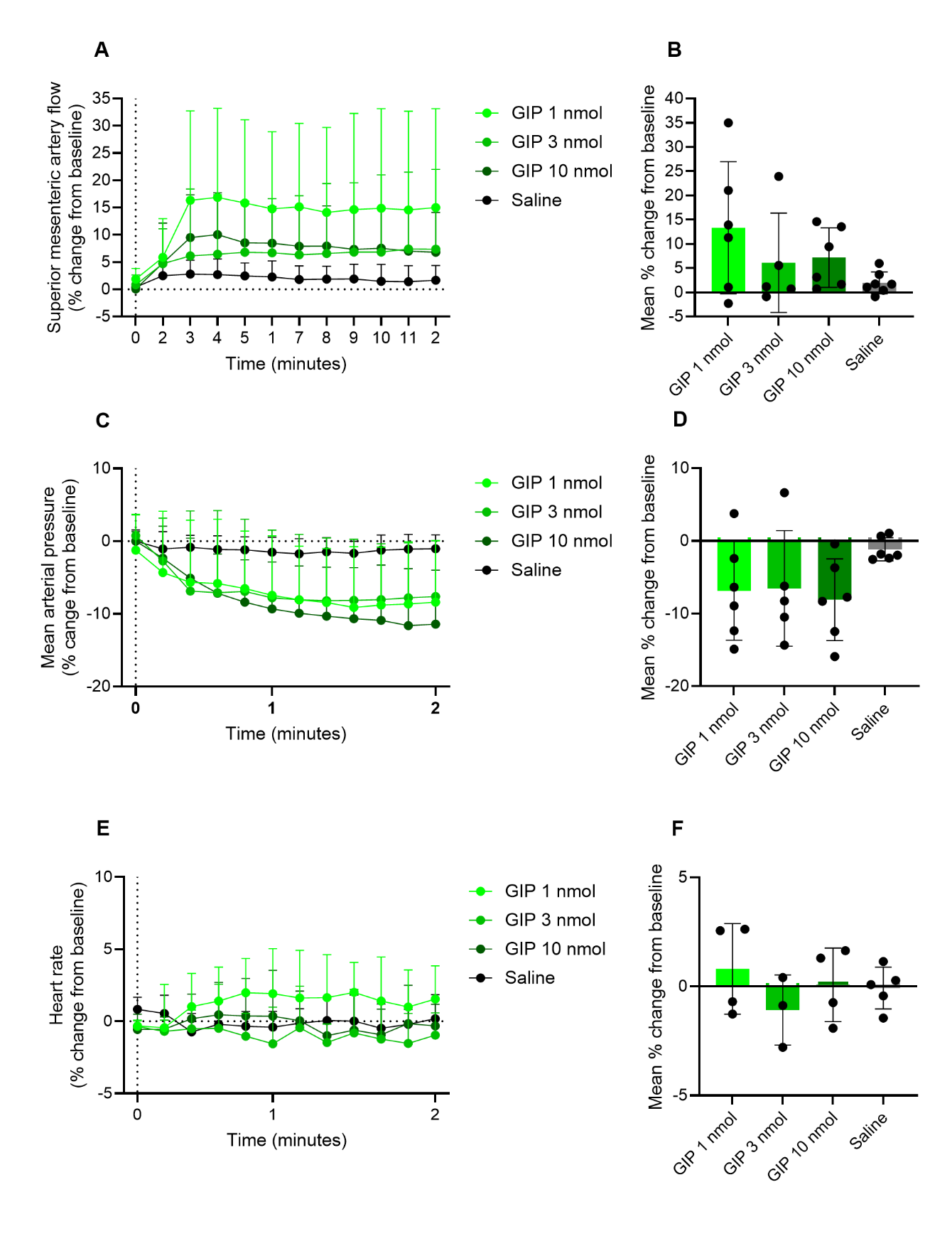


**Supplementary Figure 5.** **GIP increases superior mesenteric artery flow in rats**. Percent change from baseline in (*A* and *B*) superior mesenteric artery blood flow, (*C* and *D*) mean arterial blood pressure, and (*E* and *F*) heart rate in response to an intravenous bolus injection of 1 nmol, 3 nmol, 10 nmol GIP, and saline. P-values by ordinary one-way ANOVA, all compared to saline, corrected for multiple testing using the Dunnett test**.** Data shown as mean±SD, n=3-6. Due to the experimental setup the saline controls are the same in Supplementary Figure 3, 5, and Figure 5, and the Supplementary Figures showing the corresponding actual values (Supplementary Figure 4, 6, and 10).


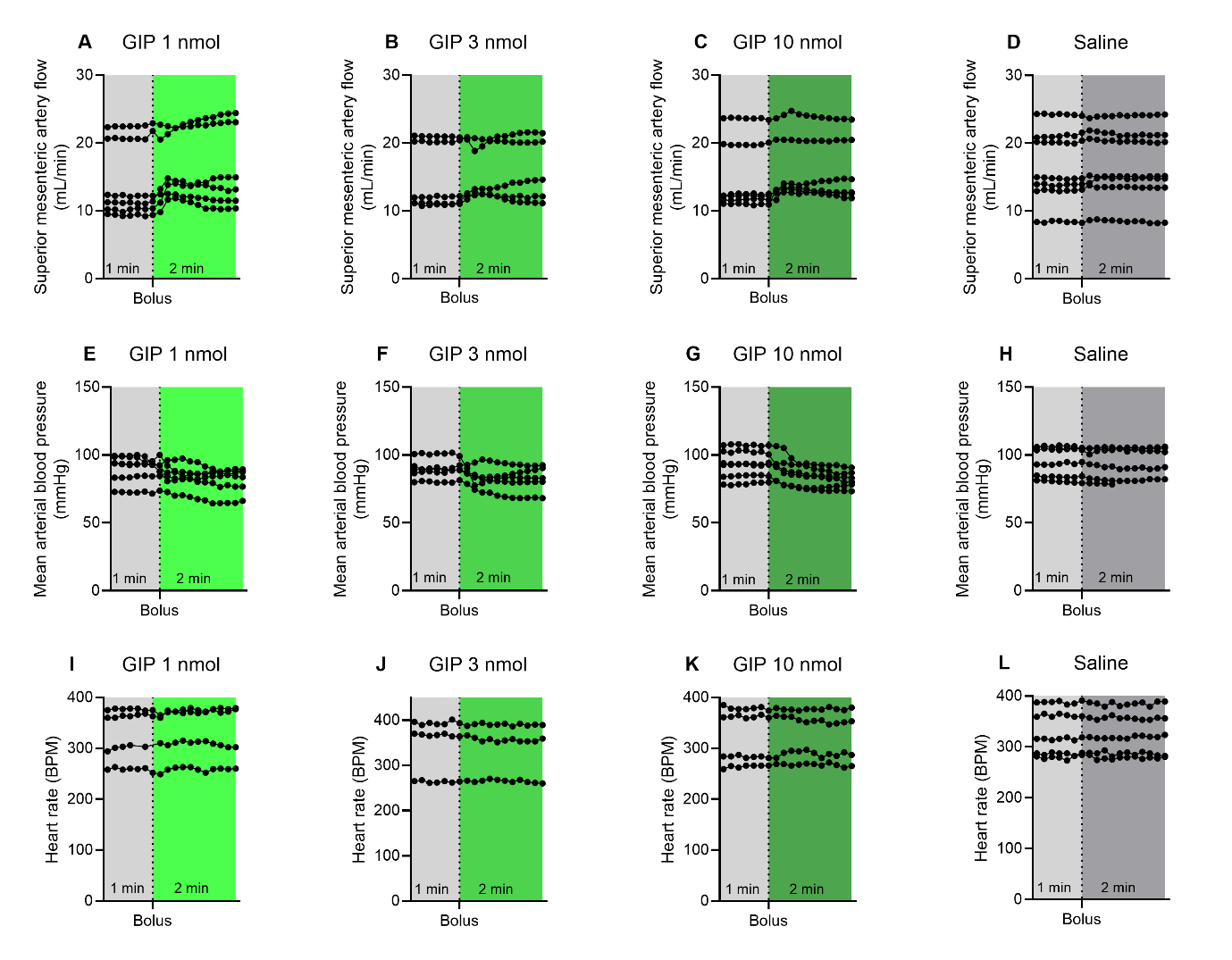


**Supplementary Figure 6. GIP increases superior mesenteric artery flow in rats**. (*A*-*D*) Superior mesenteric artery blood flow and (*E*-*H*) mean arterial blood pressure in response to an intravenous bolus injection of (*A, E*, *I*) 1 nmol, (*B, F, J*) 3 nmol, (*C, G, K*) 10 nmol GIP, and (*D, H, L*) saline. Due to the experimental setup the saline controls are the same in Supplementary Figure 3, 5, and Figure 5, and the Supplementary Figures showing the corresponding actual values (Supplementary Figure 4, 6, and 10).


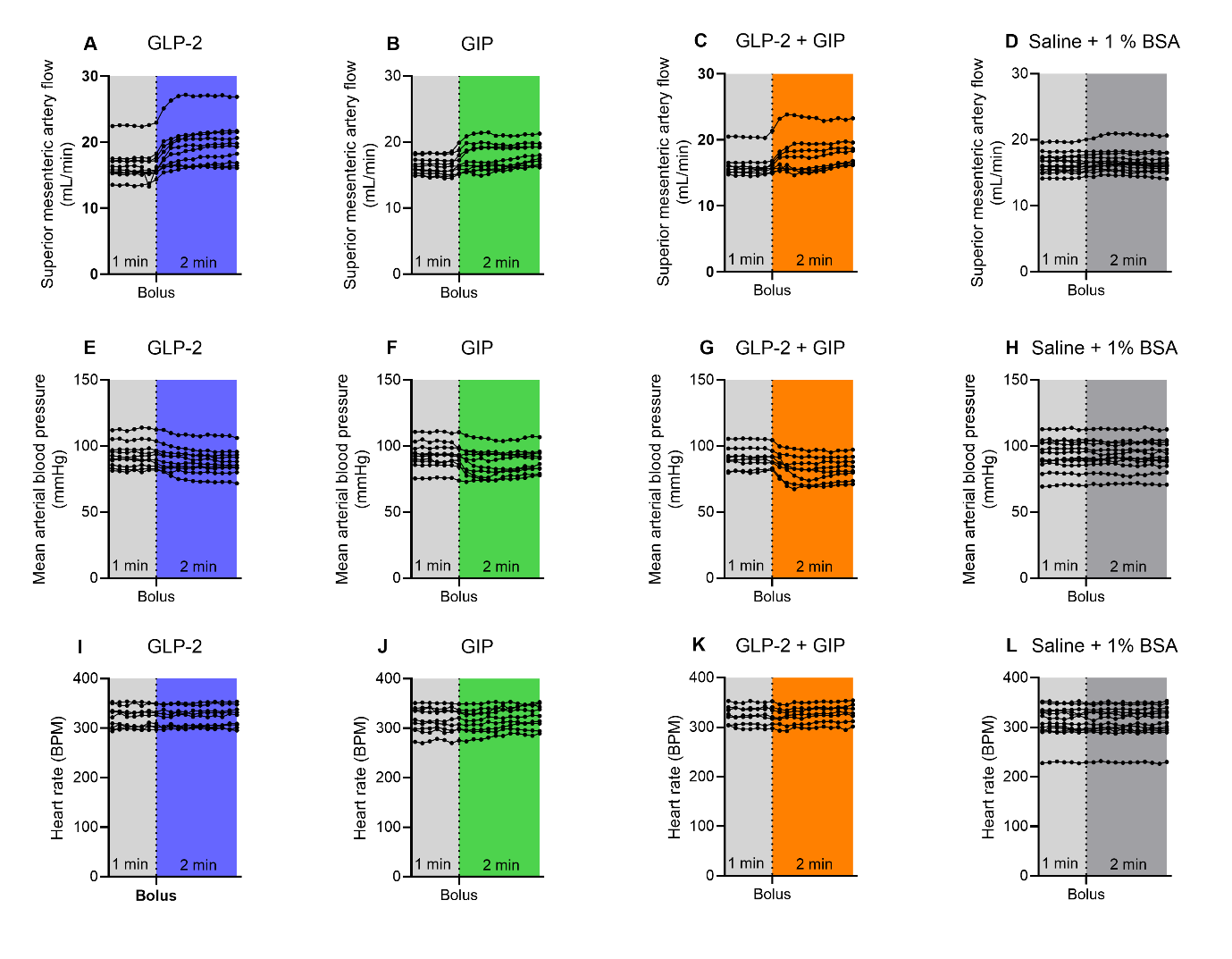

**Supplementary Figure 7. GLP-2 and GIP do not act synergistically to increase superior mesenteric blood flow**. (*A*-*D*) Superior mesenteric artery blood flow, (*E*-*H*) mean arterial blood pressure, and (*I*-*L*) heart rate in response to an intravenous bolus injection of (*A, E, I*) 0.1 nmol GLP-2, (*B, F, J*) 0.1 nmol GIP, (*C, G, K*) GLP-2 + GIP both 0.05 nmol, and (*D, H, L*) saline +1% BSA.


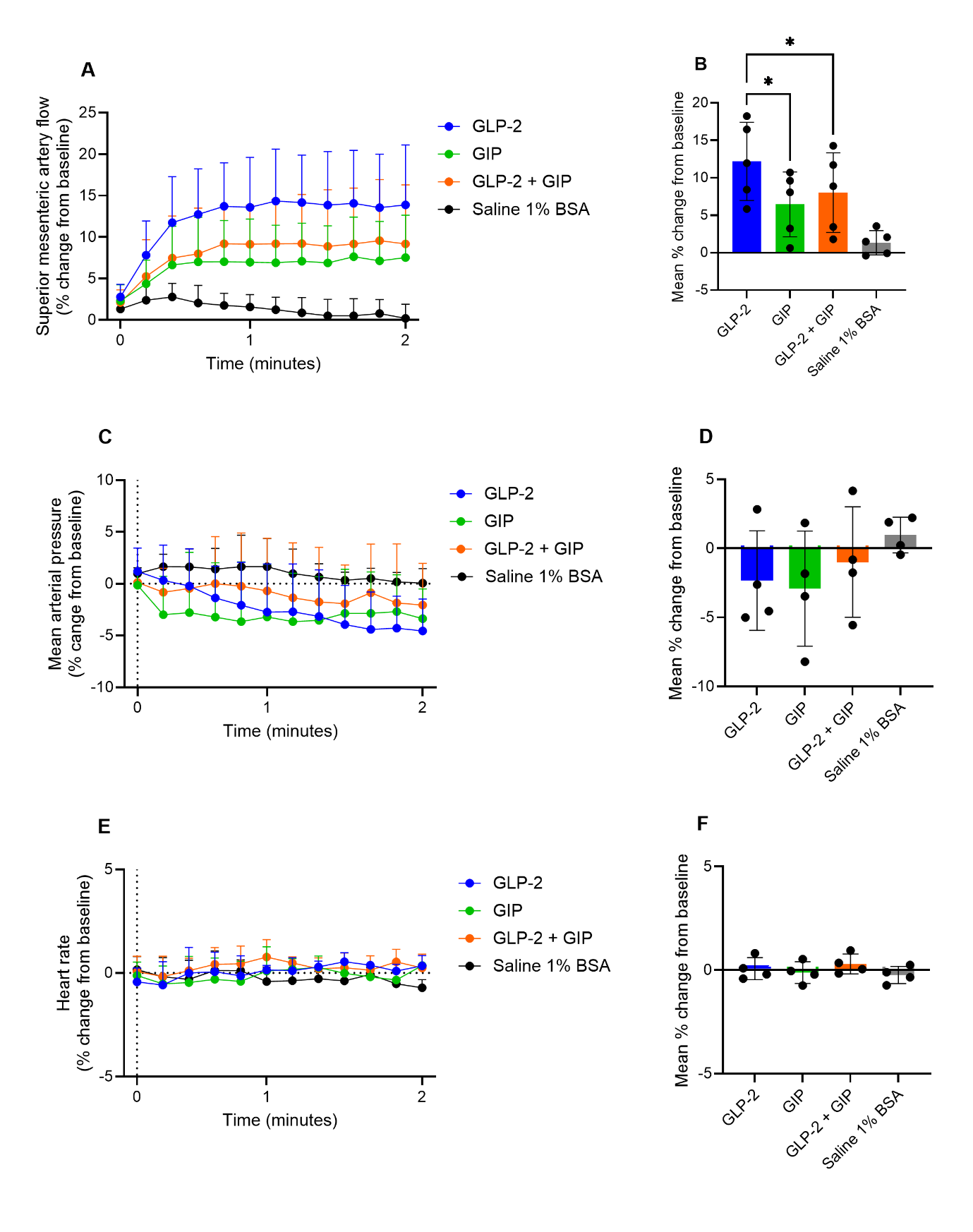
**Supplementary Figure 8**. **GLP-2 and GIP do not act additively to increase superior mesenteric blood flow**. Percent change in (*A* and *B*) **s**uperior mesenteric artery blood flow, (*C* and *D*) mean arterial blood pressure, and (*E* and *F*) heart rate in response to an intravenous bolus injection of 0.05 nmol GLP-2, 0.05 nmol GIP, GLP-2 + GIP both 0.05 nmol, and saline + 1% BSA. P-values by repeated measurement, one-way ANOVA analysis, all groups compared, corrected for multiple testing using the Tukey test**.** Data shown as mean±SD, n=4-5.


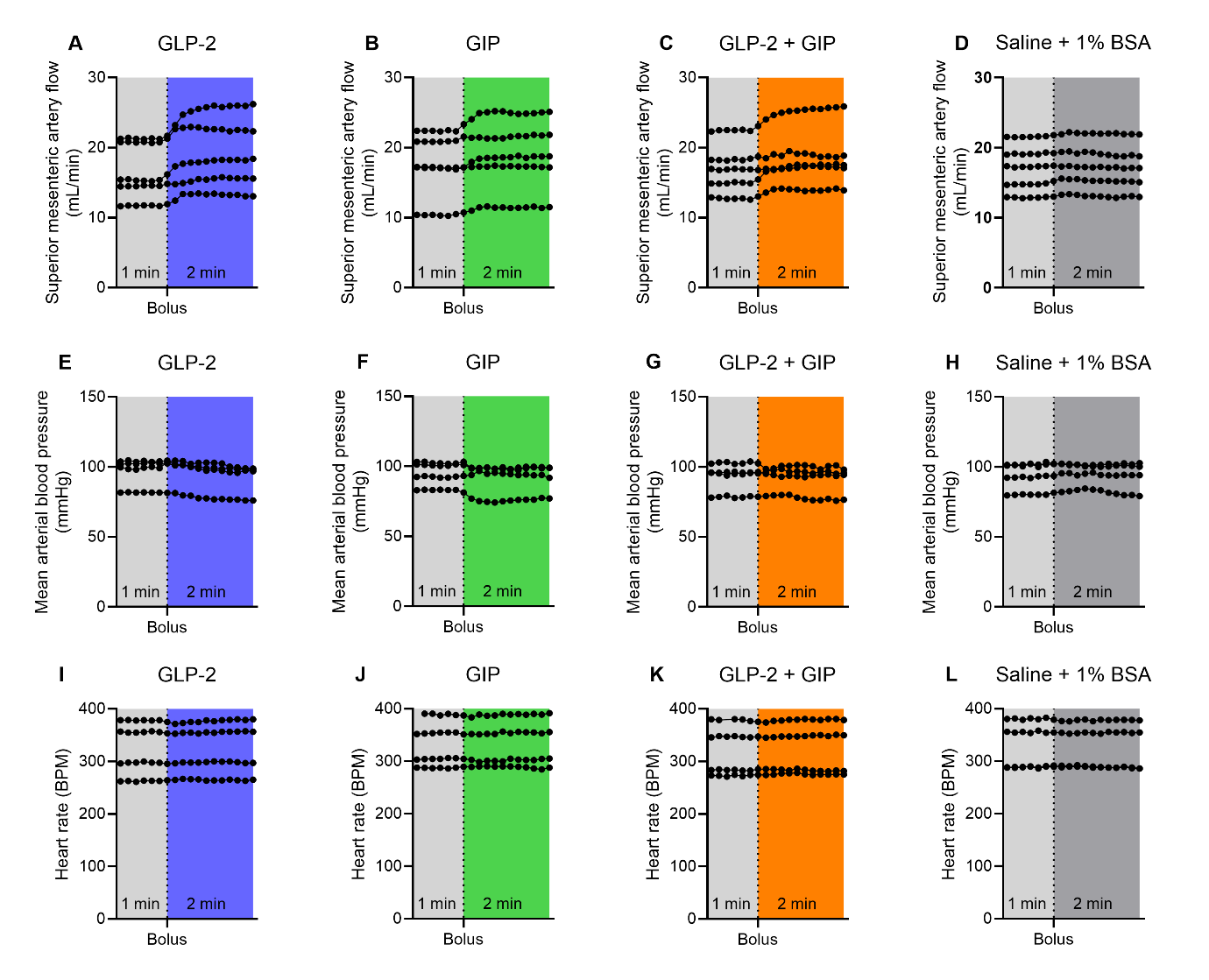


**Supplementary Figure 9**. **GLP-2 and GIP do not act additively to increase superior mesenteric blood flow**. (*A*-*D*) **S**uperior mesenteric artery blood flow, (*E*-*H*) mean arterial blood pressure, and (*I*-*L*) heart rate in response to an intravenous bolus injection of (*A*, *E*, *I*) 0.05 nmol GLP-2, (*B*, *F*, *J*) 0.05 nmol GIP, (*D*, *H*, *L*) 0.05 nmol GLP-2+0.05 nmol GIP, and (*D*, *H*, *L*) saline +1% BSA.


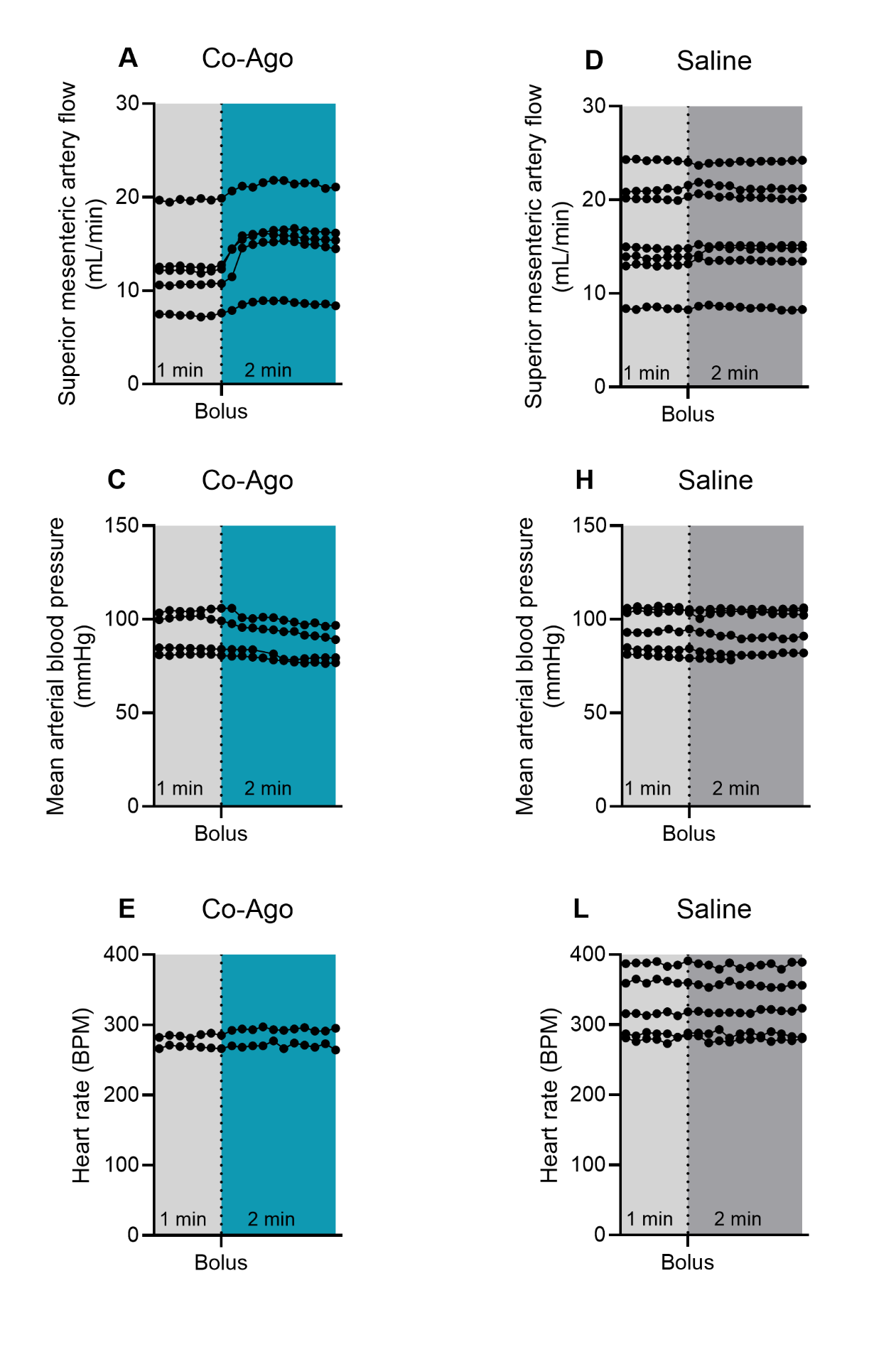


**Supplementary Figure 10.** **The GLP-2/GIP co-agonist (Co-Ago) increases superior mesenteric artery flow in rats**. (*A*-*B*) Superior mesenteric artery blood flow, (*C*-*D*) mean arterial blood pressure, and (*E* and *F*) heart rate in response to an intravenous bolus injection of (*A*, *C*, *E*) 0.2 µmol GLP-2/GIP receptor co-agonist; Co-Ago and (*B*, *D*, *F*) saline. Due to the experimental setup, the saline controls are the same in Supplementary Figure 3, 5, and Figure 5, and the Supplementary Figures showing the corresponding actual values (Supplementary Figure 4, 6, and 10).


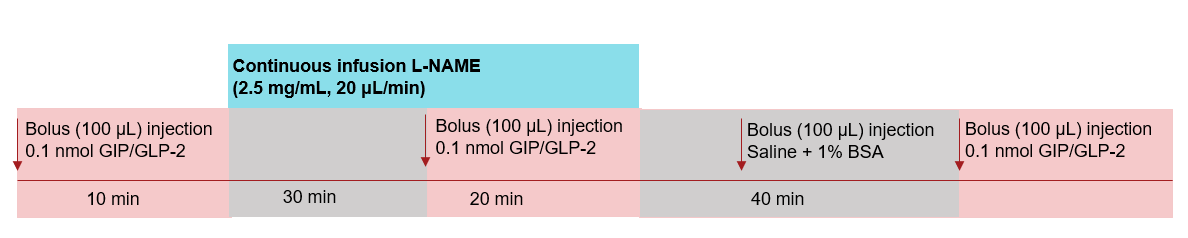


**Supplementary Figure 11**. The experimental setup when investigation the effect of L-NAME on the effect of GLP-2 and GIP on superior mesenteric artery blood flow. L-NAME was infused 30 min prior to bolus infusion of the peptides and continued for 20 min following the bolus infusion. The L-NAME infusion was stopped 40 min before the last peptide bolus infusion. The arrows indicate the bolus injections.


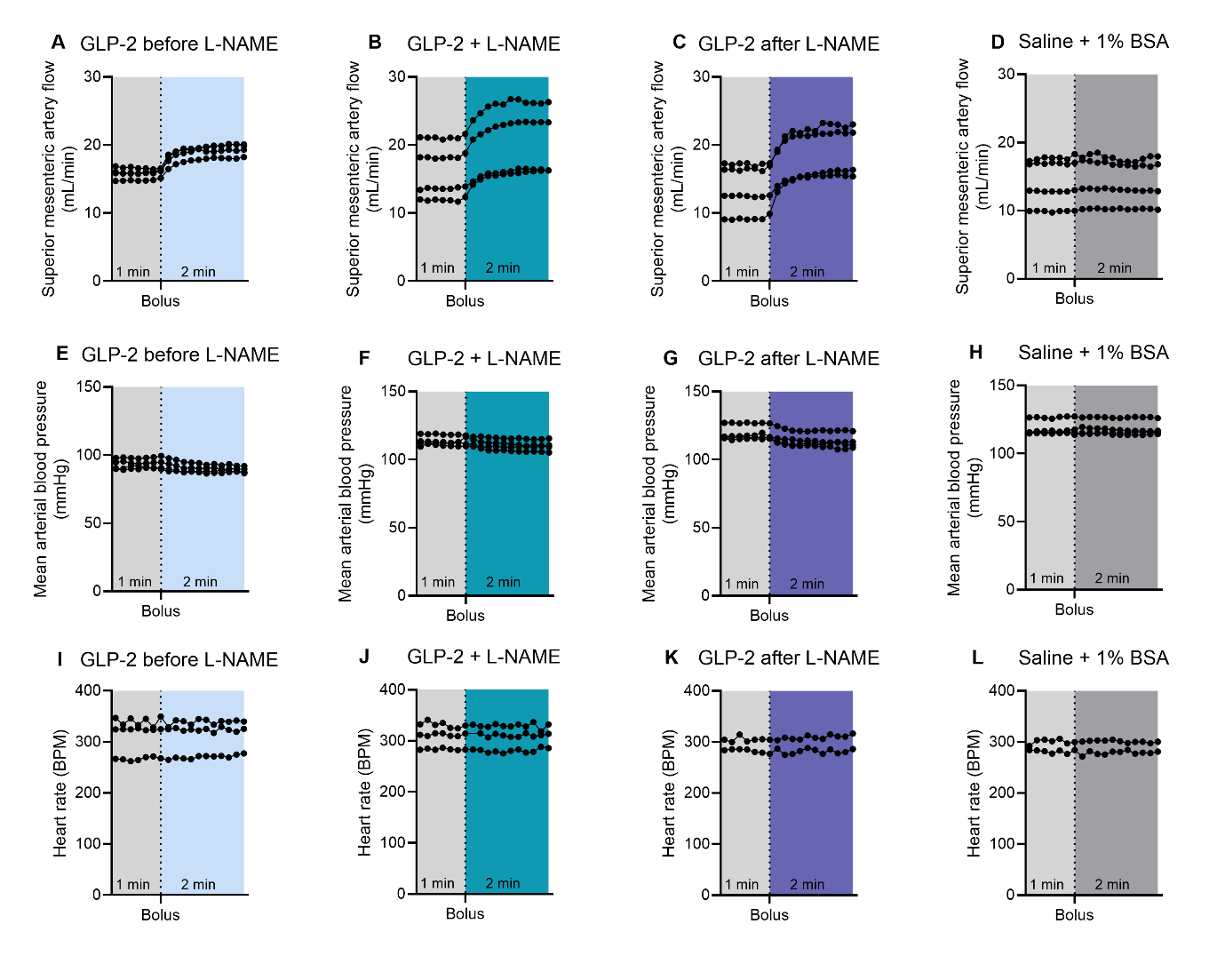


**Supplementary Figure 12.** **GLP-2 increases superior mesenteric artery blood flow in rats independent of nitric oxide.** (*A*-*D*) Superior mesenteric artery blood flow, (*E*-*H*) mean arterial blood pressure, and (*I*-*L*) heart rate in response to an intravenous bolus injection of (*A, E, I*) 0.1 nmol GLP-2, (*B, F, J*) 0.1 nmol GLP-2 during an intravenous infusion of L-NAME, (*C, G, K*) 0.1 nmol GLP-2 40 min following the termination of the L-NAME infusion, and (*D, H, L*) saline +1% BSA.


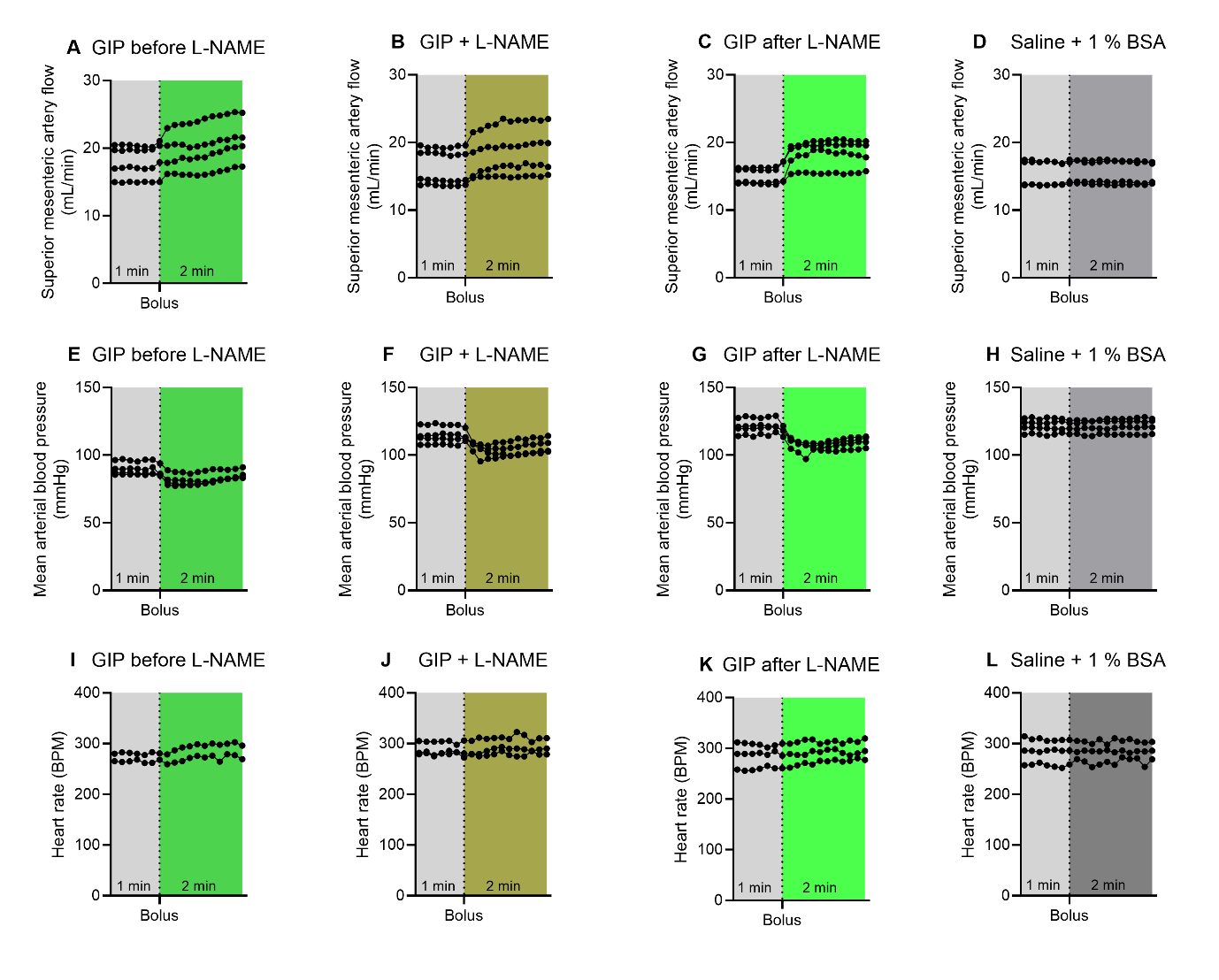
 **Supplementary Figure 13. GIP increases superior mesenteric artery blood flow in rats independent of nitric oxide.** (*A*-*D*) Superior mesenteric artery blood flow, (*E*-*H*) mean arterial blood pressure, and (*I*-*L*) heart rate in response to an intravenous bolus injection of (*A, E, I*) 0.1 nmol GIP, (*B, F, J*) 0.1 nmol GIP during an intravenous infusion of L-NAME, (*C, G, K*) 0.1 nmol GIP 40 min following the termination of the L-NAME infusion, and (*D, H, L*) saline +1% BSA.


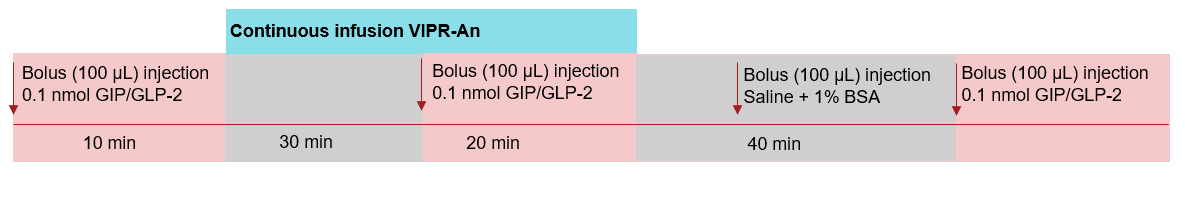


**Supplementary Figure 14**. The experimental setup when investigation the effect of a VIP receptor antagonist (VIPR-An) on the effect of GLP-2 and GIP on superior mesenteric artery blood flow. VIPR-An was infused 30 min prior to bolus infusion of the peptides and continued for 20 min following the bolus infusion. The VIPR-An infusion was stopped 40 min before the last peptide bolus infusion. The arrows indicate the bolus injections.


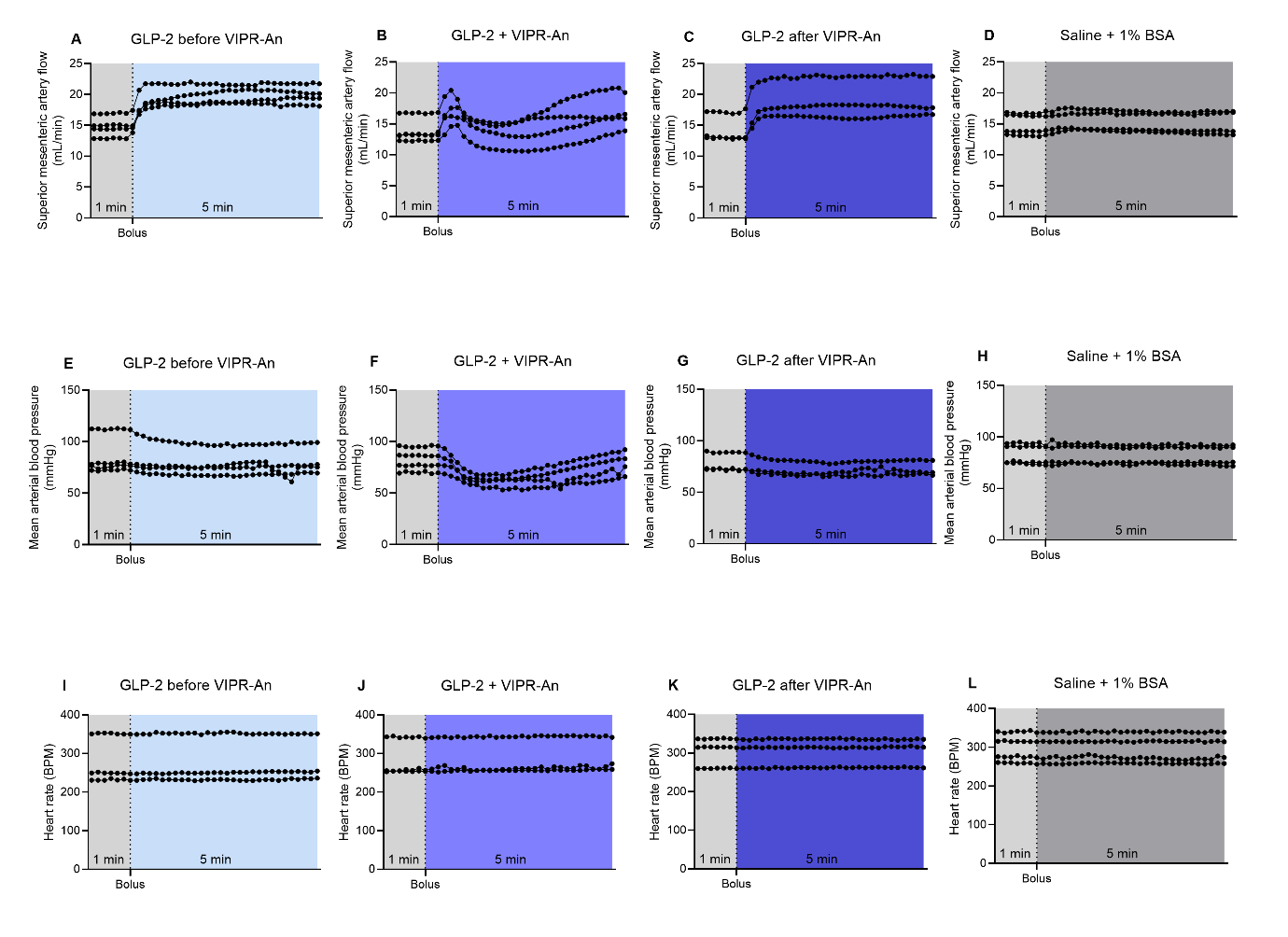


**Supplementary Figure 15. GLP-2 increases superior mesenteric artery blood flow in rats independent of VIP.** (*A*-*D*) Superior mesenteric artery blood flow, (*E*-*H*) mean arterial blood pressure, and (*I*-*L*) heart rate in response to an intravenous bolus injection of (*A, E, I*) 0.1 nmol GLP-2, (*B, F, J*) 0.1 nmol GLP-2 during an intravenous infusion of a VIP receptor antagonist (VIPR-An), (*C, G, K*) 0.1 nmol GLP-2 40 min following the termination of the VIPR-An infusion, and (*D, H, L*) saline +1% BSA.


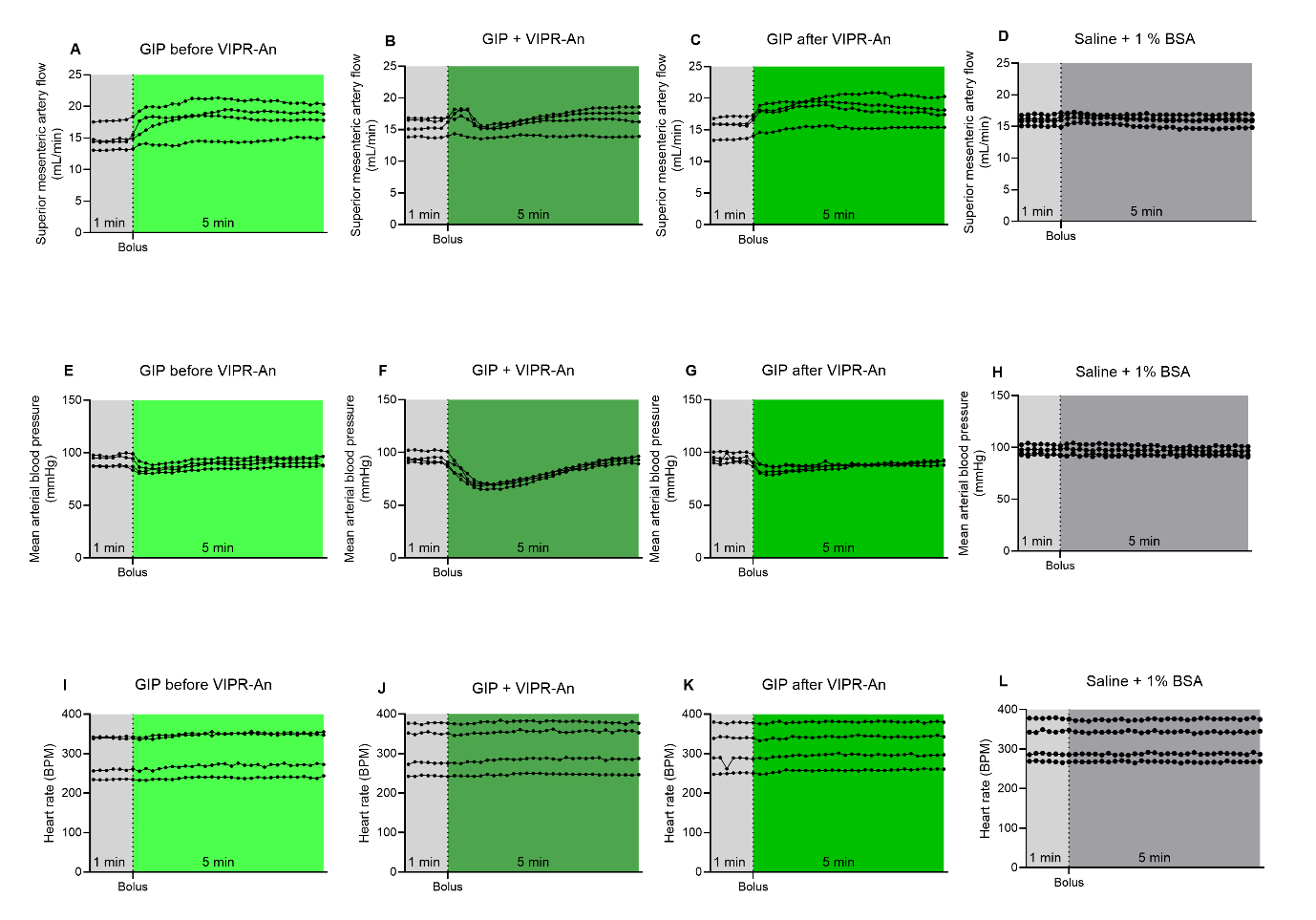


**Supplementary Figure 16. GIP increases superior mesenteric artery blood flow in rats independent of VIP.** (*A*-*D*) Superior mesenteric artery blood flow, (*E*-*H*) mean arterial blood pressure, and (*I*-*L*) heart rate in response to an intravenous bolus injection of (*A, E, I*) 0.1 nmol GIP, (*B, F, J*) 0.1 nmol GIP during an intravenous infusion of a VIP receptor antagonist (VIPR-An), (*C, G, K*) 0.1 nmol GIP 40 min following the termination of the VIPR-An infusion, and (*D, H, L*) saline +1% BSA.


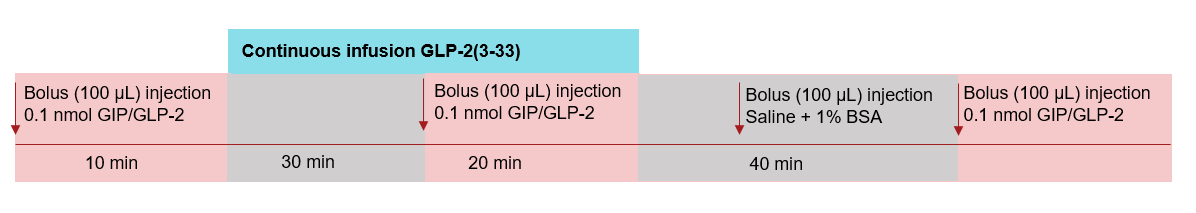


**Supplementary Figure 17**. The experimental setup when investigation the effect of GLP-2(3-33) on the effect of GLP-2 on superior mesenteric artery blood flow. GLP-2(3-33) was infused 30 min prior to bolus infusion of the peptides and continued for 20 min following the bolus infusion. The GLP-2(3-33) infusion was stopped 40 min before the last peptide bolus infusion. The arrows indicate the bolus injections.


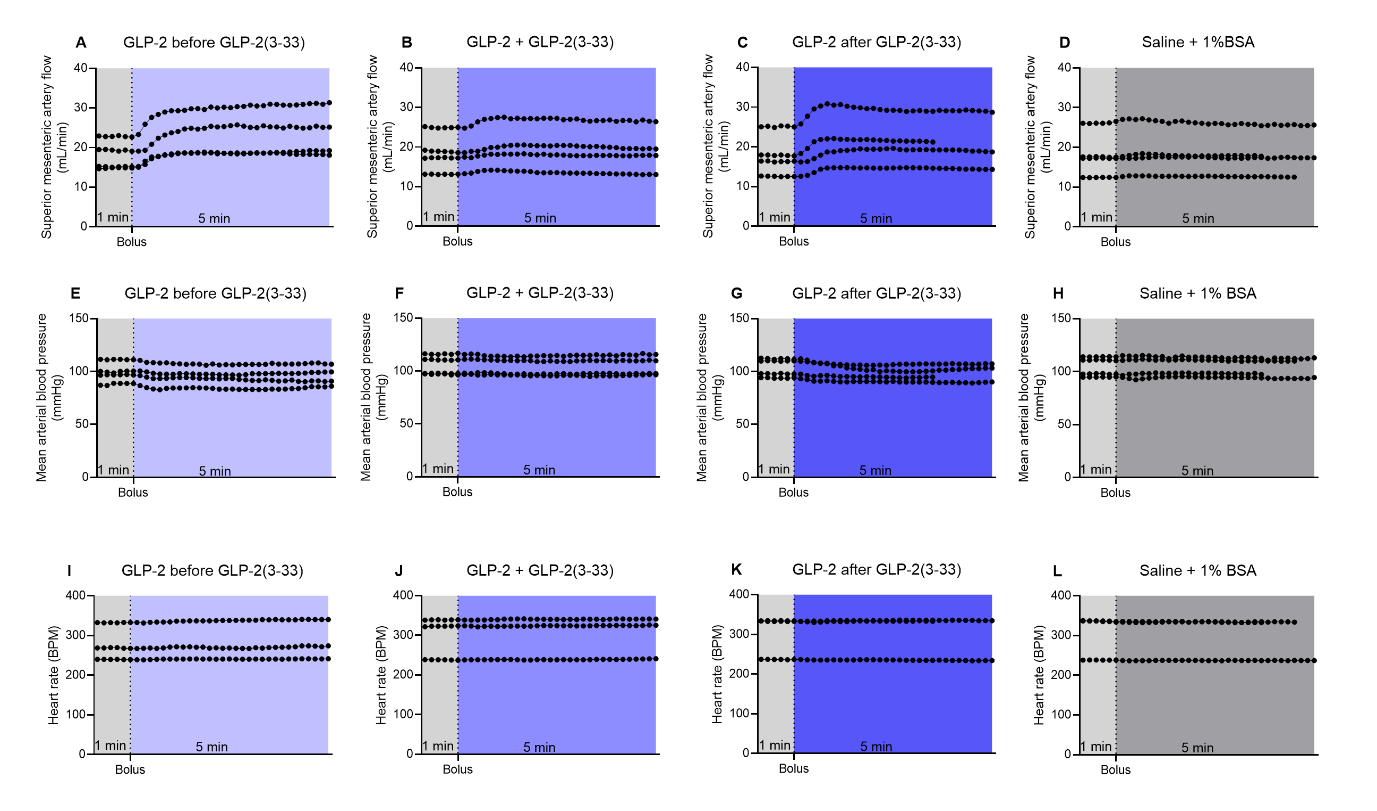


**Supplementary Figure 18. GLP-2(3-33) reduces the GLP-2-induced increase in superior mesenteric artery blood flow in rats.** (*A*-*D*) Superior mesenteric artery blood flow, (*E*-*H*) mean arterial blood pressure, and (*I*-*L*) heart rate in response to an intravenous bolus injection of (*A, E, I*) 0.05 nmol GLP-2, (*B, F, J*) 0.05 nmol GLP-2 during an intravenous infusion GLP-2(3-33), (*C, G, K*) 0.05 nmol GLP-2 after the termination of the GLP-2(3-33) infusion, and (*D, H, L*) saline +1% BSA.
